# Inhibitor-Resistant Mutants Give Important Insights into Candida albicans ABC Transporter Cdr1 Substrate Specificity and Help Elucidate Efflux Pump Inhibition

**DOI:** 10.1128/AAC.01748-21

**Published:** 2022-01-18

**Authors:** Masakazu Niimi, Kyoko Niimi, Koichi Tanabe, Richard D. Cannon, Erwin Lamping

**Affiliations:** a Sir John Walsh Research Institute, University of Otago, Dunedin, New Zealand; b Department of Food Science and Human Nutrition, Faculty of Agriculture, Ryukoku University, Shiga, Japan

**Keywords:** *Candida albicans*, Cdr1, ABC transporter, PDR transporter, drug resistance, efflux pump inhibitors, transport cycle, kinetics

## Abstract

Overexpression of ATP-binding cassette (ABC) transporters is a major cause of drug resistance in fungal pathogens. Milbemycins, enniatin B, beauvericin, and FK506 are promising leads for broad-spectrum fungal multidrug efflux pump inhibitors. The characterization of naturally generated inhibitor-resistant mutants is a powerful tool to elucidate structure-activity relationships in ABC transporters. We isolated 20 Saccharomyces cerevisiae mutants overexpressing Candida albicans ABC pump Cdr1 variants resistant to fluconazole efflux inhibition by milbemycin α25 (8 mutants), enniatin B (8), or beauvericin (4). The 20 mutations were in just 9 residues at the centers of transmembrane segment 1 (TMS1) (6 mutations), TMS4 (4), TMS5 (4), TMS8 (1), and TMS11 (2) and in A713P (3), a previously reported FK506-resistant “hot spot 1” mutation in extracellular loop 3. Six Cdr1-G521S/C/V/R (TMS1) variants were resistant to all four inhibitors, four Cdr1-M639I (TMS4) variants were resistant to milbemycin α25 and enniatin B, and two Cdr1-V668I/D (TMS5) variants were resistant to enniatin B and beauvericin. The eight milbemycin α25-resistant mutants were altered in four amino acids as follows: G521R, M639I, A713P, and T1355N (TMS11). These four Cdr1 variants responded differently to various types of inhibitors, and each exhibited altered substrate specificity and kinetic properties. The data infer an entry gate function for Cdr1-G521 and a role for Cdr1-A713 in the constitutively high Cdr1 ATPase activity. Cdr1-M639I and -T1355N possibly cause inhibitor resistance by altering TMS contacts near the substrate/inhibitor-binding pocket. Models for the interactions of substrates and different types of inhibitors with Cdr1 at various stages of the transport cycle are presented.

## INTRODUCTION

Bacteria, fungi, and human cancer cells frequently develop resistance to cytotoxic agents by overexpressing multidrug efflux pumps of the ATP-binding cassette (ABC) transporter superfamily (1). Overexpression of the pleiotropic drug resistance (PDR) ABC transporter Cdr1 of the opportunistic fungal pathogen Candida albicans protects cells from a wide range of structurally unrelated cytotoxic agents, including azole antifungals (2). ABC transporters are one of the largest protein superfamilies that are found in all kingdoms of life (3, 4). Eukaryotic ABC proteins can be divided into nine major subfamilies (ABCA, ABCB, ABCC, ABCD, ABCE, ABCF, ABCG, ABCH, and ABCI) based on their phylogeny, topology, and structure (5–7). Seven of these subfamilies (ABCA, ABCB, ABCC, ABCD, ABCG, ABCH, and ABCI) are transporters with transmembrane domains (TMDs) attached to their nucleotide binding domains (NBDs) (5, 7). Plants (7) and fungi (8–11) typically have a much larger arsenal of ABC transporters than mammals. Yet, despite their obvious importance for the proper development and survival of multicellular organisms, in most cases, very little is known about their biological function or the complete range of their substrates. Cdr1 is a fungal PDR transporter (12) of the ABCG subfamily. Unlike all other ABC transporters, ABCG transporters have an inverted topology with the two NBDs preceding their respective TMDs [(NBD-TMD)_2_] (2). The two cytosolic NBDs bind and hydrolyze ATP which are thought to trigger large conformational changes at the TMDs and the extrusion of substrates through a centrally located substrate and inhibitor binding pocket that provides a pathway for substrates to exit the transporter.

Significant progress has been made recently in determining the structure of important ABC transporters, including the major human multidrug efflux pumps ABCB1 (13) and ABCG2 (14, 15) and the human cholesterol transporter ABCG5-G8 (16), with the first ABCG transporter structure being published in 2016. Since then, a number of additional ABCG2 structures have been solved, in both the open (15) and closed (14) conformation. These structures have provided useful templates to generate models of the prototype fungal PDR transporters C. albicans Cdr1 and Saccharomyces cerevisiae Pdr5 (17). Although progress has also been made in understanding the structure-activity relationship for some efflux pump inhibitors of human ABCG2 (18), much remains to be discovered about the substrate specificity and inhibitor susceptibility of the vast ABCG transporter family. For example, it remains largely unknown why some ABCG transporters have a very narrow substrate range (e.g., cholesterol transporter ABCG5-G8) while others (e.g., multidrug efflux pump ABCG2) can transport a large array of unrelated compounds. Understanding how PDR transporters function, how they select and transport substrates, and how they are inhibited by different types of inhibitors will be important to combat the efflux-mediated drug resistance of plant and human fungal pathogens.

Over the past two decades, we have developed and refined a yeast-based efflux pump expression system (9, 11, 19–22) that allows us to study fungal efflux pumps in great detail. The overexpression of functional efflux pumps in the genetically modified host S. cerevisiae AD1-8u^-^ has many advantages, of which one is the ability to isolate naturally selected inhibitor-resistant efflux pump mutants. This approach has the advantage of unbiased sampling of the entire landscape of possible mutations that can overcome efflux pump inhibition. Using this approach, we isolated and characterized 72 FK506- (17) and 12 RC21v3-resistant (23) mutants of C. albicans Cdr1 and S. cerevisiae Pdr5. The 72 FK506-resistant isolates overexpressing Cdr1/Pdr5 had mutations in 40 different residues concentrated in 2 major hot spots within or near the extracellular domain (ED) of Cdr1/Pdr5, of which about half were in “hot spot 1” in, or proximal to, A713/A723 in the extracellular loop 3 (EL3) of the ED and T540/T550 and S542/T552 in EL1 (17). In addition, all 12 RC21v3-resistant Cdr1 variants had mutations in just 5 residues of the ED (23). We hypothesized that FK506 is a competitive efflux pump inhibitor of Cdr1 and Pdr5 (17) and RC21v3 binds to the ED of Cdr1 and “freezes” the transporter in either the open or closed conformation (23). A comprehensive review of fungal efflux pump inhibitors was provided recently by Monk and Keniya (24).

Milbemycins are 16-membered ring macrolides with broad-spectrum acaricidal and insecticidal activities. Their low toxicity to mammals and plants, possibly because they are substrates of most plant and vertebrate P-glycoproteins (25, 26), has made them one of the most rapidly expanding classes of insecticides. Milbemycins are effective inhibitors of fungal PDR transporters (20, 23, 27, 28) and are promising leads for combination therapy with azoles against azole-resistant fungal pathogens. Azoles in combination with milbemycin A3/A4 oxime derivatives have been used successfully to treat mice with systemic infections caused by azole-resistant clinical C. albicans or Candida glabrata isolates (29). Another important class of broad-spectrum fungal efflux pump inhibitors is the depsipeptides enniatin B (20, 21, 28, 30) and beauvericin (22, 28, 31). Despite their importance, little is known about how these inhibitors interact with, and inhibit, fungal multidrug efflux pumps. The isolation of 20 variants overexpressing Cdr1 mutated in just 9 residues that were resistant to the inhibition of fluconazole (FLC) efflux by milbemycin α25 (8 isolates; 4 different mutants in 4 residues), enniatin B (8 isolates; 8 mutants in 7 residues), or beauvericin (4 isolates; 4 mutants in 2 residues) and the detailed characterization of the four most prominent mutations now provide important clues about the structure-activity relationship of Cdr1 substrates and inhibitors. Tentative models of how different classes of compounds inhibit Cdr1 efflux pump function and how Cdr1 differentiates between substrates and inhibitors are discussed.

## RESULTS

### Isolation of milbemycin α25-resistant Cdr1 mutants.

**FIG 1:**
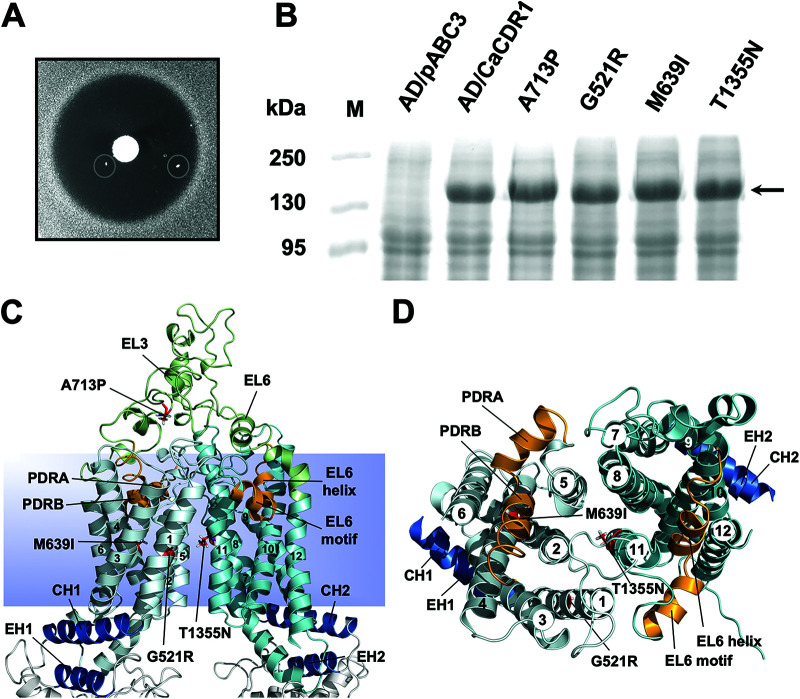
Isolation of milbemycin α25-resistant FLC efflux pump mutants of S. cerevisiae AD1-8u^-^ cells overexpressing C. albicans *CDR1*. (A) Individual colonies (circled) resistant to milbemycin α25 appeared within the growth inhibitory zones surrounding a milbemycin α25 (5 μg) disc placed on YPD agar medium containing 0.25 MIC (128 μg/mL) of FLC after 7 days of incubation at 30°C. (B) Cdr1 expression in AD/CDR1, milbemycin α25-resistant mutants (G521R, A713P, M639I, and T1355N), and the negative-control strain AD/pABC3. Plasma membrane preparations (30 μg) isolated from logarithmic-phase cells were separated by 8% SDS-PAGE and visualized with Coomassie brilliant blue R-250 staining. The arrow indicates the ∼170-kDa Cdr1 protein band. (C and D) Cartoon models of the TMDs of the inward-open conformation of Cdr1 (17) with TMS1-12 numbered 1 to 12. TMD1 is light blue, TMD2 is cyan, and EL3 and EL6 are green. The coupling helices (CH1 and CH2) and the E-helices (EH1 and EH2) that connect the TMDs to the NBDs are blue, and the residues causing milbemycin α25 resistance, namely G521R (TMS1), M639I (TMS4), A713P (EL3) and T1355N (TMS11), are shown as red sticks. The images in C and D show the TMDs viewed from the side (C) or the top (D) with EL3 and EL6 and the NBDs removed in D for clarity. The PDR transporter-defining motifs (orange) that dip halfway down into the TMDs of the transporter fit tightly between TMS2, -4, -5, and -6 (i.e., PDRA-PDRB) and TMS8, -10, -11, and -12 (i.e., EL6 helix-EL6 motif), respectively.

S. cerevisiae colonies overexpressing Cdr1 that were resistant to milbemycin α25 appeared at low frequency (∼10^−6^ to 10^−7^) in growth inhibitory zones around discs containing milbemycin α25 on yeast extract-peptone-dextrose (YPD) agar plates with high, but sub-MICs of FLC (Fig. 1A). Of 10 milbemycin α25-resistant AD/CDR1 isolates, 8 had acquired single point mutations in the *CDR1* open reading frame (ORF) that caused 4 different amino acid substitutions (Table 1; see Table S1 in the supplemental material). The other two isolates (R25-4 and R25-10) were discarded because they apparently had mutations outside the *CDR1* ORF. The A713P and M639I mutations were each found on three separate occasions, and G521R and T1355N were found once each. The three independent M639I suppressor mutants contained two different nucleotide changes (G1917C in isolate R25-3 and G1917A in isolates R25-8 and R25-9) (Table S1). The four milbemycin α25 resistance mutations were in the large extracellular loop 3 (EL3; A713P) and near the centers of TMS1 (G521R), TMS4 (M639I), and TMS11 (T1355N) (Fig. 1C and D). Plasma membrane fractions separated by SDS-PAGE showed that the Cdr1 expression levels in the mutants were equal to the expression level of wild type (wt) Cdr1 (Fig. 1B; see Fig. S1A in the supplemental material). A list of S. cerevisiae strains used in this study is provided in Table 1. We designated the four milbemycin α25-resistant Cdr1 mutants G521R, M639I, A713P, and T1355N.

**TABLE 1:** Saccharomyces cerevisiae strains used in this study

Strain^*a*^	Genotype or description	Source
AD1-8u^-^	*MATα, PDR1-3, ura3, his1, Δyor1*::*hisG, Δsnq2*::*hisG, Δpdr10*::*hisG, Δpdr11*::*hisG, Δycf1*::*hisG, Δpdr3*::*hisG, Δpdr15*::*hisG, Δpdr5*::*hisG*	34
AD/pABC3	AD1-8u^-^, *Δpdr5*::pABC3 (empty vector control)	20
AD/CDR1	AD1-8u^-^, *Δpdr5*::pABC3-CDR1B	20
AD/CDR1-G521R	Milbemycin α25-resistant isolate R25-2	This study
AD/CDR1-G521R	Enniatin B-resistant isolate EN-1	This study
AD/CDR1-G521V	Enniatin B-resistant isolate EN-2	This study
AD/CDR1-G521S	Beauvericin-resistant isolate BE-1	This study
AD/CDR1-G521C	Beauvericin-resistant isolate BE-2	This study
AD/CDR1-G521V	Beauvericin-resistant isolate BE-3	This study
AD/CDR1-M639I	Milbemycin α25-resistant isolate R25-3	This study
AD/CDR1-M639I	Milbemycin α25-resistant isolate R25-8	This study
AD/CDR1-M639I	Milbemycin α25-resistant isolate R25-9	This study
AD/CDR1-M639I	Enniatin B-resistant isolate EN-3	This study
AD/CDR1-L664I	Enniatin B-resistant isolate EN-4	This study
AD/CDR1-L665S	Enniatin B-resistant isolate EN-5	This study
AD/CDR1-V668I	Enniatin B-resistant isolate EN-6	This study
AD/CDR1-V668D	Beauvericin-resistant isolate BE-4	This study
AD/CDR1-A713P	Milbemycin α25-resistant isolate R25-1	This study
AD/CDR1-A713P	Milbemycin α25-resistant isolate R25-6	This study
AD/CDR1-A713P	Milbemycin α25-resistant isolate R25-7	This study
AD/CDR1-F1235V	Enniatin B-resistant isolate EN-7	This study
AD/CDR1-T1355N	Milbemycin α25–resistant isolate R25-5	This study
AD/CDR1-M1356I	Enniatin B-resistant isolate EN-8	This study

a Mutant strains are listed in order according to where in the molecule their *CDR1* mutations occurred and in which inhibitor-resistance screen they were isolated.

### G521R and A713P have significantly altered substrate specificities.

**FIG 2:**
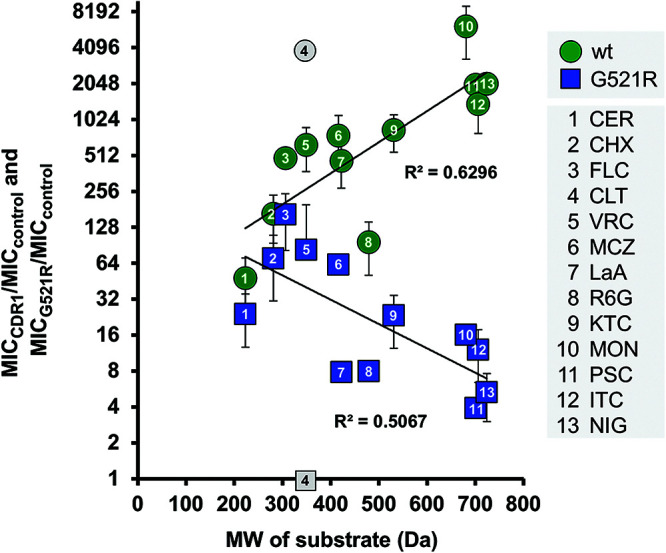
The size-dependent substrate transport of wt Cdr1 was inverted in G521R. The fold-reduced MICs in G521R (relative to AD/CDR1) and the molecular weights (MWs) of the 13 test substrates (Fig. S1) are shown in Table 2. The test substrates were numbered 1 to 13 from smallest to largest MW. A graph of the fold increased drug resistance (*y* axis; 1 = no drug transport), relative to the susceptible AD/pABC3 control strain, plotted against the MW of the test compounds revealed a size-dependent phenotype for wt Cdr1 (green circles), which was inverted in G521R (blue squares). The results are the means ± SD of 2 to 7 independent experiments.

If milbemycin α25 inhibits FLC efflux by binding to the substrate binding pocket, milbemycin α25-resistant Cdr1 mutants could exhibit altered substrate specificities. Therefore, the MICs of 13 efflux pump substrates with different molecular weights (MWs; 223 to 724 Da) and diverse chemistries (see Fig. S2 in the supplemental material) were determined for AD/pABC3, AD/CDR1, and the four milbemycin α25-resistant mutants (see Table S2 in the supplemental material). The MICs were determined to be an indirect measure of the ability of Cdr1 to efflux the substrates. As expected, all mutants effluxed FLC to some degree (as they were isolated on FLC-containing media), although A713P and T1355N had 2-fold and G521R had 4-fold reduced minimum growth inhibitory concentrations of FLC (MIC_FLC_) compared with wt Cdr1 (Table 2). S. cerevisiae cells overexpressing M639I and T1355N had unchanged, or at worst 4-fold reduced, MICs for all test substrates. The only exception was posaconazole (PSC), for which the MIC was 16-fold reduced in both mutants, but this finding may be an artifact due to its low water solubility. Significant changes in substrate specificities, however, were observed for A713P. The mutant was modestly (2- to 4-fold reduced MICs) affected in the transport of FLC, voriconazole (VRC), cerulenin (CER), cycloheximide (CHX), and latrunculin A (LaA); it had an unchanged MIC for rhodamine 6G (R6G) and monensin (MON), but it was severely (8- to 32-fold reduced MICs) affected in the transport of clotrimazole (CLT), miconazole (MCZ), ketoconazole (KTC), PSC, itraconazole (ITC), and nigericin (NIG) (Table 2). G521R exhibited the most interesting phenotype. Its substrate transport profile was clearly affected in a size-dependent manner. The MICs of the smallest substrates (MWs of ≤306 Da; CER, CHX, and FLC) were 2- to 4-fold reduced; the MICs of the medium-sized (MWs of 349 to 531) substrates VRC, MCZ, KTC, LaA, and R6G were 8- to 64-fold reduced; and the MICs of the largest (MWs of >681) substrates PSC, ITC, MON, and NIG were most severely reduced (128- to 512-fold) (Table 2). The only exception was CLT, which is by definition a small substrate (MW of 345 Da). It was the only substrate whose transport was abrogated completely in G521R (gray square, Fig. 2). A graph of the fold-increased MIC values, relative to the sensitive control strain AD/pABC3, plotted against the MW of the test substrates revealed more effective transport (R^2^ = 0.63) of larger compounds by Cdr1, a trend that was reversed completely (R^2^ = 0.51) for G521R (Fig. 2). The 8-fold and 2-fold reduced MICs for R6G of G521R and M639I (Table 2) were reflected in significantly reduced R6G efflux pump activities of intact cells (Fig. S1B).

**TABLE 2:** Xenobiotic resistance of S. cerevisiae AD1-8u^-^ strains overexpressing milbemycin α25-resistant Cdr1 mutants relative to AD/CDR1^*a*^

Strain^*b*^	Fold-reduced resistance levels of:												
	Azoles^*c*^							Other xenobiotics^*c*^					
	FLC 306	CLT 345	VRC 349	MCZ 416	KTC 531	PSC 701	ITC 706	CER 223	CHX 281	LaA 422	R6G 479	MON 681	NIG 724
G521R	4	4,096	8	16	64	512	128	2	2	64	8	256	512
M639I	1	1	1	4	2	16	2	2	2	1	2	1	1
A713P	2	8	2	8	8	32	32	2	2	4	1	1	16
T1355N	2	2	1	4	2	16	2	2	1	4	1	1	1

a The maximum fold-reduced resistance levels (i.e., no transport) equaled the drug susceptibilities of the sensitive control strain AD/pABC3. They were 512 (FLC), 4,096 (CLT), 512 (VRC), 1,024 (MCZ), 1,024 (KTC), 2048 (PSC), 1,024 (ITC), 32 (CER), 128 (CHX), 512 (LaA), 64 (R6G), 4,096 (MON), and 2,048 (NIG), respectively (1 = no change).

b G521R, AD/CDR1-G521R; M639I, AD/CDR1-M639I; A713P, AD/CDR1-A713P; T1355N, AD/CDR1-T1355N.

c Cdr1 drug substrates are listed according to their molecular weight from lowest (left) to highest (right). Numbers underneath each compound are the molecular weights in Da.

### Inhibitor susceptibilities of milbemycin α25-resistant Cdr1 mutants.

**FIG 3:**
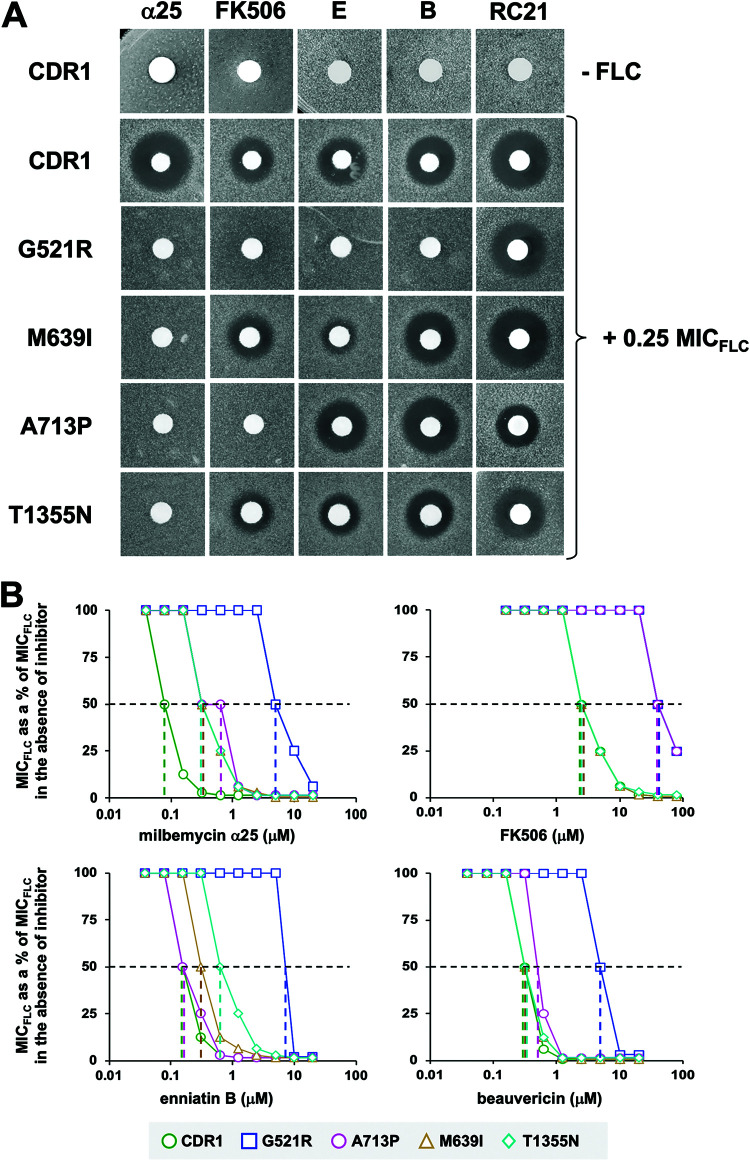
FLC chemosensitization of S. cerevisiae AD1-8u^-^ overexpressing wt Cdr1 and milbemycin α25-resistant Cdr1 mutants. (A) YPD agar plates with or without 0.25 MIC_FLC_ were seeded with AD1-8u^-^ cells overexpressing wt Cdr1 (CDR1) or milbemycin α25-resistant Cdr1 mutants (G521R, M639I, A713P, and T1355N). Filter discs were loaded with 5 μg milbemycin α25 (α25), 10 μg FK506, 0.2 μg enniatin B (E), 0.5 μg beauvericin (B), or with 6 nmol RC21v3 (RC21). The top row is the inhibitor-only control (i.e., YPD agar plates without FLC) which demonstrates that the five inhibitors alone are not toxic to cells overexpressing wt Cdr1. (B) Two-dimensional checkerboard assays agreed with the results of the agarose disc diffusion chemosensitization assays. Graphs of MIC_FLC_s of S. cerevisiae AD1-8u^-^ cells overexpressing wt Cdr1 (green circles), G521R (blue squares), M639I (orange triangles), A713P (pink circles), or T1355N (cyan diamonds) as a percentage of the MIC_FLC_ in the absence of inhibitor, plotted against increasing concentrations of the indicated efflux pump inhibitors. Dashed horizontal lines indicate 50% inhibition, and colored vertical dashed lines indicate the IC_50_ values (Table 4) for the corresponding Cdr1 variants.

Agar diffusion FLC chemosensitization assays were used to test the susceptibilities of the milbemycin α25-resistant mutants to the Cdr1 inhibitors milbemycin α25, FK506, enniatin B, beauvericin, and RC21v3 (Fig. 3A; see Fig. S3 in the supplemental material). None of the inhibitors, at the concentrations tested, were toxic to S. cerevisiae AD/CDR1 (Fig. 3A, top row) or AD/pABC3 (17), although a slight growth inhibitory zone was visible for AD/CDR1 around the milbemycin α25 disc (Fig. 3A). Indeed, no milbemycin α25 toxicity was detected in liquid MIC assays for any of the Cdr1 variants, or AD/pABC3, even at the highest test concentration of 20 μM (11.4 mg/L) (Table 3). We also detected no synergy between FK506, enniatin B, or beauvericin with the two Cdr1 efflux pump substrates FLC and R6G in AD/pABC3 (32). Thus, the synergies for the five inhibitor/FLC combinations of AD/CDR1 and the Cdr1 variants (Fig. 3A) at the concentrations used are most likely due to specific interactions of these compound combinations with the substrate binding pocket of Cdr1. All inhibitors exquisitely chemosensitized (i.e., they inhibited the FLC transport of) cells overexpressing wt Cdr1 to 0.25× its MIC_FLC_ (Fig. 3A, second row). As expected, the FLC transport of all four mutants was more resistant to milbemycin α25 (which was used to select the mutants), and they remained susceptible to RC21v3, to various degrees (Fig. 3A). G521R was also resistant to FK506, enniatin B, and beauvericin (Fig. 3A). The inhibitor susceptibilities of M639I and T1355N were quite similar. As expected, both showed most resistance to milbemycin α25. They also showed substantial resistance to enniatin B but remained susceptible to FK506 (Fig. 3A). The only difference between them was that T1355N was also slightly more resistant to beauvericin and RC21v3. As expected from our previous study (17), A713P was resistant to FK506 as well as to milbemycin α25. In addition, it was also slightly resistant to RC21v3, but it remained susceptible to enniatin B and beauvericin (Fig. 3A).

**TABLE 3:** FLC chemosensitization and FICI values for four efflux pump inhibitors of AD1-8u^-^ cells overexpressing wt Cdr1 or milbemycin α25-resistant Cdr1 mutants^*a*^

Strain^*b*^	MIC_FLC_ (mg/L)	Milbemycin α25^*c*^		FK506^*c*^		Enniatin B		Beauvericin	
		MIC_FLC_^*d*^	FICI^*e*^	MIC_FLC_^*d*^	FICI^*e*^	MIC_FLC_^*d*^	FICI^*e*^	MIC_FLC_^*d*^	FICI^*e*^
CDR1	640	5 (0.63)	**<0.02**	5 (40)	**<0.26**	5 (1.25)	**<0.13**	5 (1.25)	**<0.13**
G521R	160	20 (20)	0.63	40 (80)	0.75	160 (5)	1.5	80 (5)	1.00
M639I	640	5 (2.5)	**<0.07**	5 (40)	**<0.26**	20 (1.25)	**0.16**	5 (2.5)	**<0.26**
A713P	320	5 (2.5)	**<0.08**	80 (80)	0.75	5 (1.25)	**<0.14**	5 (1.25)	**<0.14**
T1355N	320	5 (2.5)	**<0.08**	5 (40)	**<0.27**	20 (2.5)	**0.31**	5 (1.25)	**<0.14**

a The 2-dimensional checkerboard assays were performed with 2-fold serial dilutions of FLC (5 to 640 mg/L) in one dimension and 2-fold serial dilutions (0.039 to 20 μM) of milbemycin α25, enniatin B, and beauvericin or 2-fold serial dilutions (0.156 to 80 μM) of FK506 in the second dimension. The MICs of enniatin B and beauvericin were 10 μM for all strains, including AD/pABC3.

b Strain names are as shown in Table 2.

c Milbemycin α25 and FK506 alone did not inhibit the growth of any strain, including AD/pABC3, even at the highest concentrations tested. For the purpose of calculating FICI values, we assumed MICs for milbemycin α25 and FK506 of 40 and 160 μM, respectively.

d The numbers in parentheses are the lowest inhibitor concentrations (μM) necessary to achieve the lowest MIC_FLC_s, listed to the left.

e FICI values of ≤0.5 indicate synergy and are highlighted in bold text, FICI values of >0.5 to ≤1 indicate an additive effect, and FICI values of >1 indicate no difference between two drug combinations.

These results were confirmed with quantitative 2-dimensional checkerboard liquid MIC assays. Assuming MIC_FLC_ can be taken as a proxy for FLC efflux, a graph of the reduction in MIC_FLC_ with increasing inhibitor concentrations (Fig. 3B) allowed an estimation of the 50% inhibitory concentration (IC_50_) values for FLC efflux inhibition in whole cells (Table 4). Milbemycin α25, FK506, enniatin B, and beauvericin sensitized AD/CDR1 (green circles, Fig. 3B) to FLC concentrations (5 mg/L) that were comparable to the MIC_FLC_ (1 mg/L) of AD/pABC3. The concentration required to inhibit FLC transport (i.e., reduce the MIC_FLC_) of AD/CDR1 by 50% (i.e., IC_50_ of MIC_FLC_) (Table 4) varied significantly for the four efflux pump inhibitors. Milbemycin α25 was the most potent inhibitor of FLC efflux of wt Cdr1 (IC_50_ of 0.08 μM) followed by enniatin B (0.16 μM), beauvericin (0.31 μM), and FK506 (2.5 μM). G521R was 20 to 100 times more resistant to all four inhibitors (Table 4). M639I and T1355N were ∼5 times more resistant to milbemycin α25, 2 (M639I) and 4 times (T1355N) more resistant to enniatin B, and susceptible to FK506 and beauvericin. A713P was 5 and 16 times more resistant to milbemycin α25 and FK506, respectively, although it was almost as susceptible to enniatin B and beauvericin as wt Cdr1 (Table 4). Strong synergies (bold highlighted in Table 3) with fractional inhibitory concentration indexes (FICIs) of <0.26 were detected for all four inhibitor/FLC combinations with wt Cdr1. The greatest synergy (FICI of 0.02) was for the milbemycin α25/FLC combination (Table 3). Even though the FLC transport of all four mutants was 5 to 100 times more resistant to milbemycin α25, only G521R failed to show synergy (FICI to 0.63) (Table 3) for this drug combination. Interestingly, there was also no synergy for any other inhibitor with FLC (FICIs of 0.75 to 1.5) for cells overexpressing G521R (Table 3). A713P was the only other Cdr1 mutant that showed a loss of synergy (FICI of 0.75) for one inhibitor/FLC combination (FK506/FLC) (Table 3). The other mutants showed slightly reduced synergies of inhibitors with FLC (Table 3).

**TABLE 4:** Inhibitor sensitivities of FLC efflux of live whole cells and the *in vitro* ATPase activities of wt Cdr1 and milbemycin α25-resistant Cdr1 mutants

Strain^*a*^	IC_50_^*c*^ (μM)											
	α25^*b*^		FK506		Enniatin B		Beauvericin		Oligomycin		Vanadate	
	MIC_FLC_	ATPase	MIC_FLC_	ATPase	MIC_FLC_	ATPase	MIC_FLC_	ATPase	MIC_FLC_	ATPase	MIC_FLC_	ATPase
CDR1	0.08	2.8	2.5	1.25	0.16	0.18	0.31	0.24	ND^*d*^	1.2	ND	1.2
G521R	8	28	40	14.5	8	4.0	6	2.8	ND	3.1	ND	>20
M639I	0.4	30	2.5	0.8	0.31	0.5	0.31	0.15	ND	1.0	ND	1.8
A713P^*e*^	0.4	>30	40	>20	0.16	>30	0.63	>30	ND	0.9	ND	0.26
T1355N	0.4	6.1	2.5	1.0	0.63	0.04	0.31	0.06	ND	0.54	ND	6.0

a Strain names are as shown for Table 2.

b α25, milbemycin α25.

c The IC_50_ values for the inhibition of FLC efflux (i.e., MIC_FLC_) and the Cdr1 ATPase activities were calculated from Fig. 3B and 4, respectively.

d ND, not determined.

e High concentrations of milbemycin α25, enniatin B, and beauvericin induced the ATPase activity of Cdr1-A713P.

### Effects of efflux pump inhibitors on the ATPase activity of wt and milbemycin α25-resistant Cdr1 mutants.

The effects of pump inhibitors on the Cdr1 ATPase activities of wt Cdr1 and the four milbemycin α25-resistant mutants G521R, M639I, A713P, and T1355N were measured (Fig. 4). IC_50_ values for the inhibition of the ATPase activities by milbemycin α25, FK506, enniatin B, and beauvericin were calculated from the data in Fig. 4 and are presented in Table 4 next to their IC_50_ values for the inhibition of FLC efflux. For Cdr1, G521R, M639I, and T1355N, there was a good correlation between the IC_50_ values for FLC efflux inhibition and the IC_50_ values for the inhibition of the ATPase activities by FK506, enniatin B, and beauvericin (R^2^ = 0.99; A713P was excluded because the ATPase IC_50_ values could not be measured accurately) (see Fig. S4 in the supplemental material). There was no such correlation, however, for milbemycin α25 (Table 4 and Fig. S4).

**FIG 4:**
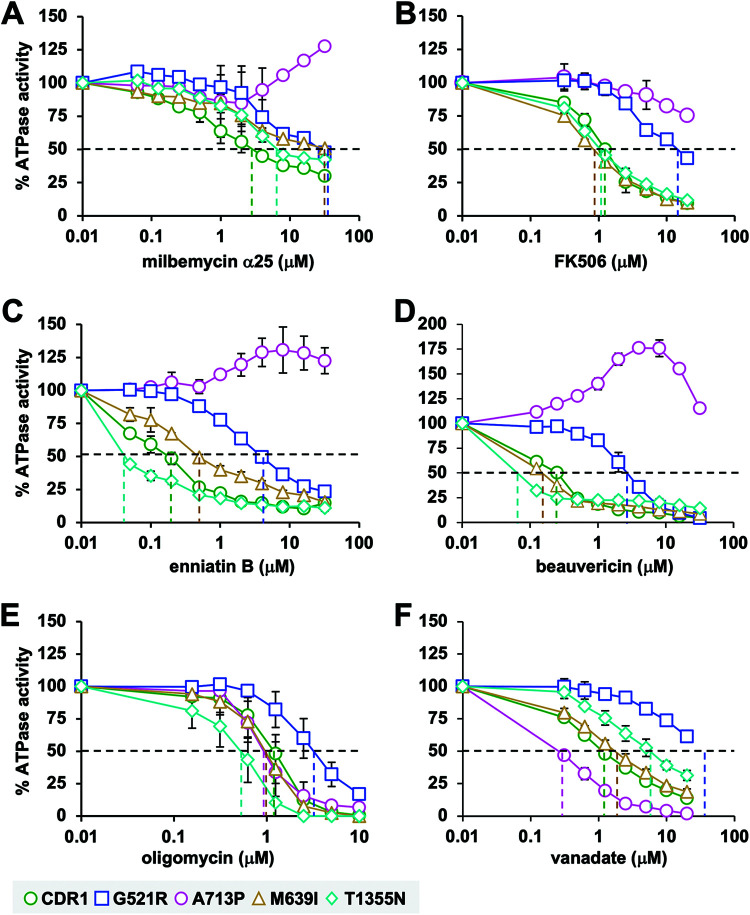
Effect of pump inhibitors on the Cdr1 ATPase activities of wild-type Cdr1 (green circles) and milbemycin α25-resistant mutants G521R (blue squares), M639I (orange triangles), A713P (pink circles), and T1355N (cyan diamonds). The inhibitor sensitivities of the ATPase activities of wt Cdr1 and the milbemycin α25-resistant mutants were determined for milbemycin α25 (A), FK506 (B), enniatin B (C), beauvericin (D), oligomycin (E), and vanadate (F). The Cdr1 ATPase activities are shown as the percentages of the ATPase activities in the absence of inhibitor. Values are the means (± SD) of three independent experiments. The IC_50_ values for the various efflux pump inhibitors are presented in Table 4. They are the inhibitor concentrations where the vertical dashed lines intersect with the *x* axis.

The ATPase activities of all four Cdr1 mutants were more resistant to milbemycin α25, and G521R and A713P had dramatically increased resistance to all four efflux pump inhibitors (Fig. 4A to D and Table 4). The IC_50_ values for the inhibition of the wt Cdr1 ATPase activity by FK506 (1.25 μM), enniatin B (0.18 μM), and beauvericin (0.24 μM) were comparable to the IC_50_ values for the inhibition of FLC efflux by whole cells (Table 4). Milbemycin α25 was, however, an exception as it inhibited the FLC efflux of wt Cdr1 at 35 times lower concentrations (0.08 μM) than it inhibited the ATPase activity (2.8 μM) (Table 4). Similar discrepancies in the milbemycin α25 susceptibilities were detected in all other Cdr1 mutants (Table 4 and Fig. S4). The differences between the two IC_50_ values for milbemycin α25 were more pronounced in wt Cdr1 (35 times), M639I (75 times), and A713P (>75 times) than those in T1355N (15 times) or G521R (3.5 times) (Table 4). These differences are most likely caused by the presence of FLC in the whole-cell FLC transport inhibition assay. Kueppers, et al. provided evidence for a possible “cross talk” between a substrate (R6G) and an efflux pump inhibitor (FK506) in the S. cerevisiae efflux pump Pdr5, in which R6G interfered with the binding of FK506 to the substrate binding pocket of the FK506-resistant Pdr5-S1360F mutant (33). The presence of FLC in the substrate binding pocket may also have interfered with the inhibitory action of enniatin B and beauvericin in T1355N, although in that case, the presence of FLC actually reduced the inhibition of enniatin B and beauvericin compared to their effect on the ATPase activity of T1355N (Table 4). Even though enniatin B and beauvericin appeared to have ∼4 times higher affinities to the substrate binding pocket of T1355N than wt Cdr1 (i.e., their IC_50_ values for the ATPase activity were ∼4 times lower than those for wt Cdr1), the presence of FLC impaired this interaction in T1355N so that the FLC efflux pump function of T1355N remained equally susceptible to beauvericin and was even ∼4 times more resistant to enniatin B than wt Cdr1 (Table 4). The response of the A713P ATPase activity to the four efflux pump inhibitors was even more unexpected. The FLC efflux pump function of A713P was 5 and 16 times more resistant to milbemycin α25 and FK506 and as susceptible to enniatin B and beauvericin as wt Cdr1 (Table 4). Its ATPase activity, however, was not inhibited at all at lower concentrations and even slightly induced (∼20% to 75%) at higher (∼10 μM) milbemycin α25, enniatin B, and beauvericin concentrations, although it was still partially inhibited at high (20 μM) FK506 concentrations (Fig. 4A to D). Yet, for reasons explained below, the FLC efflux pump function of A713P remained exquisitely sensitive to enniatin B and beauvericin, which is similar to wt Cdr1 (Table 4). There were no substantial differences between the FK506, enniatin B, and beauvericin susceptibilities (i.e., the IC_50_ values) of the FLC efflux pump function and the ATPase activity of M639I and the FK506 susceptibilities of T1355N (Table 4).

### Effects of oligomycin and vanadate on the ATPase activities of wt and milbemycin α25-resistant Cdr1 mutants.

Unlike most ABC proteins, the ATPase activity of PDR transporters is oligomycin sensitive, and its oligomycin sensitivity is used to determine PDR transporter-specific ATPase activities in crude plasma membrane preparations (20). Vanadate is a Pi analogue that binds tightly to catalytically active NBDs and inhibits the ATP hydrolysis of ABC transporters (34–38). We noticed little variation in the oligomycin susceptibilities of the ATPase activities of wt Cdr1 and the four milbemycin α25-resistant mutants. There were no significant changes to the oligomycin sensitivities of Cdr1 in M639I and A713P (Fig. 4E and Table 4). However, the ATPase activity of G521R was ∼3 times more resistant and that of T1355N was ∼2 times more sensitive to oligomycin than wt Cdr1 (Table 4). To investigate the possible effects of the various milbemycin α25-resistant mutations on their posthydrolytic Cdr1 conformations, we also tested their vanadate sensitivities. The vanadate sensitivity of M639I was practically the same as that of wt Cdr1 (Fig. 4F and Table 4). However, A713P was 5 times more sensitive and T1355N was 5 times more resistant to vanadate than wt Cdr1, and the ATPase activity of G521R was almost completely resistant to vanadate (Fig. 4F and Table 4).

### Kinetic parameters of the ATPase activities of wt and milbemycin α25-resistant Cdr1 mutants.

To understand the possible modulations of the Cdr1 ATPase activities in the milbemycin α25-resistant mutants, their kinetic parameters alone and in response to various Cdr1 efflux pump inhibitors were determined. Unfortunately, there was insufficient compound to determine the kinetic parameters in response to milbemycin α25. The *K_m_* (0.62 mM), *V*_max_ (303 nmol/min/mg), and *k*_cat_ (8.6 s^−1^) values of the ATPase activity of wt Cdr1 (Table 5) were comparable to those reported previously for Cdr1 (39) and Pdr5 (40). The *K_m_* values of M639I (0.69 mM) and T1355N (0.58 mM) were comparable to those of wt Cdr1 but the *V*_max_ values of M639I (233 nmol/min/mg) and T1355N (189 nmol/min/mg) were reduced by ∼20% and ∼40%, respectively (Table 5). The kinetic parameters of A713P and G521R were more severely affected. The respective *K_m_* (0.35 mM), *V*_max_ (101 nmol/min/mg), and *k*_cat_ (2.9 s^−1^) values for A713P were ∼40%, ∼70%, and ∼70% lower, respectively, than those for wt Cdr1, and the *k*_cat_/*K_m_* ratio (8.3 s^−1^ mM^−1^), an indicator of catalytic efficiency, was also decreased to ∼60% of wt Cdr1 (13.9 s^−1^ mM^−1^) (Table 5). This result may explain the ∼2- to 4-fold reduced pumping efficiencies of A713P for most of the smaller Cdr1 efflux pump substrates (i.e., FLC, VRC, CER, CHX, LaA, and R6G) (Table 2). In contrast to A713P, the *K_m_* (0.92 mM), *V*_max_ (497 nmol/min/mg), and *k*_cat_ (14.1 s^−1^) values of G521R were ∼50% to 65% higher than those in wt Cdr1, although the overall catalytic efficiency (*k*_cat_/*K_m_*, 15.4 s^−1^ mM^−1^) was similar to that of wt Cdr1 because the reduced affinity of ATP for the NBD (*K_m_*, 0.92 mM) was compensated for by an increased turnover rate (*k*_cat_, 14.1 s^−1^) (Table 5).

**TABLE 5:** Kinetic parameters of the ATPase activities of wt Cdr1 and milbemycin α25-resistant Cdr1 mutants

Strain	*K_m_* (mM)	*V*_max_ (nmol/min/mg)	*k*_cat_^*a*^ (s^−1^)	*k*_cat_/*K_m_* (s^−1^mM^−1^)
CDR1	0.62 ± 0.04	303 ± 10	8.6 ± 0.27	13.9 ± 0.8
G521R	0.92 ± 0.06	497 ± 61	14.1 ± 1.72	15.4 ± 2.2
M639I	0.69 ± 0.12	233 ± 38	6.6 ± 1.07	9.7 ± 1.5
A713P	0.35 ± 0.03	101 ± 2	2.9 ± 0.04	8.3 ± 0.7
T1355N	0.58 ± 0.08	189 ± 20	5.4 ± 0.65	9.3 ± 1.3

a To calculate enzyme turnover rates (*k*_cat_) and enzyme efficiencies (*k*_cat_/*K_m_*), we assumed that 10% of the plasma membrane protein was Cdr1 (MW of 169,941 Da). The values presented are the means (± SD) of at least three independent experiments.

### Effects of inhibitors on the kinetic properties of the Cdr1 ATPase activities, and dissociation constant (*K_i_*) values for the inhibitors.

**FIG 5:**
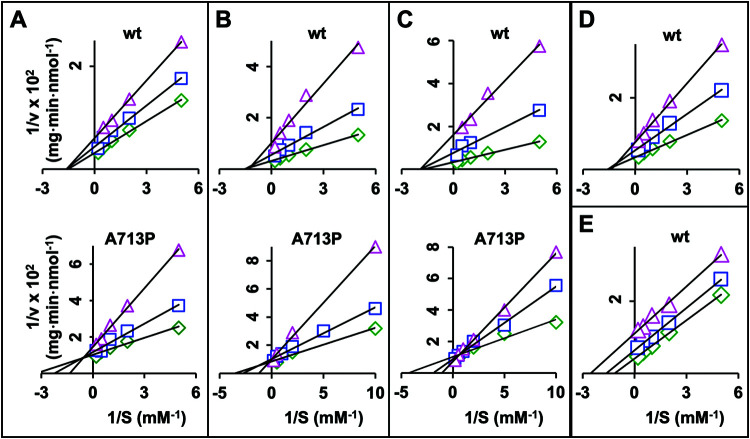
Lineweaver-Burk plots of the ATPase activities of wt Cdr1 and A713P in response to increasing inhibitor concentrations. (A) Top, wt Cdr1 with FK506. Concentrations were 0 (green diamond), 0.5 μM (blue square), and 1 μM (magenta triangle). Bottom, A713P with FK506. Concentrations were 0 (green diamond), 1 μM (blue square), and 10 μM (magenta triangle). (B) Top, wt Cdr1 with enniatin B. Concentrations were 0 (green diamond), 0.1 μM (blue square), and 0.5 μM (magenta triangle). Bottom, A713P with enniatin B. Concentrations were 0 (green diamond), 0.1 μM (blue square), and 10 μM (magenta triangle). (C) Top, wt Cdr1 with beauvericin. Concentrations were 0 (green diamond), 0.2 μM (blue square), and 0.5 μM (magenta triangle). Bottom, A713P with beauvericin. Concentrations were 0 (green diamond), 0.1 μM (blue square), and 2 μM (magenta triangle). (D) wt Cdr1 with oligomycin. Concentrations were 0 (green diamond), 0.5 μM (blue square), and 1 μM (magenta triangle). (E) wt Cdr1 with vanadate. Concentrations were 0 (green diamond), 0.5 μM (blue square), and 2 μM (magenta triangle).

To gain further insights into the possible modulations of the Cdr1 ATPase activities by the different efflux pump inhibitors, their effects on the kinetic properties of ATP-hydrolysis in wt Cdr1 and the milbemycin α25-resistant mutants were investigated at inhibitor concentrations that only partially inhibited the ATPase activities. The inhibition of the ATPase activities followed Michaelis-Menten kinetics. Lineweaver-Burk plots for the different types of inhibitors and examples of the most characteristic changes to the ATPase activities are presented in Fig. 5. The *K_m_* and *V*_max_ values for the Cdr1 variants in the absence and presence of the different inhibitor concentrations were calculated (Table 6).

**TABLE 6:** *K_m_* and *V*_max_ values of the ATPase activities of wild-type Cdr1 and milbemycin α25-resistant Cdr1 mutants in the presence of increasing inhibitor concentrations^*a*^

Inhibitor	Data for strain:														
	CDR1			G521R			M639I			A713P			T1355N		
	[I]	*K_m_*	*V*_max_	[I]	*K_m_*	*V*_max_	[I]	*K_m_*	*V*_max_	[I]	*K_m_*	*V*_max_	[I]	*K_m_*	*V*_max_
FK506	0	0.66	**313**	0	0.94	**556**	0	0.71	**278**	0	0.37	**101**	0	0.63	**213**
	0.5	0.63	**233**	1	0.95	**500**	1	0.66	**164**	1	0.45	**88**	1	0.59	**98**
	1.0	0.61	**164**	10	0.72	**256**	10	0.64	**123**	10	0.74	**69**	10	0.63	**61**
Enniatin B	0	0.66	**313**	0	0.85	**500**	0	0.53	**204**	0	0.31	99	0	0.47	**166**
	0.1	0.66	**182**	0.5	0.92	**385**	0.1	0.54	**164**	0.1	0.34	95	0.1	0.60	**92**
	0.5	0.76	**98**	10	0.93	**179**	0.5	0.61	**123**	10	0.81	100	0.5	0.51	**19** ^*b*^
Beauvericin	0	0.58	**303**	0	0.94	**556**	0	0.53	**204**	0	0.37	103	0	0.47	**167**
	0.2	0.53	**132**	0.1	1.21	**526**	0.1	0.53	**143**	0.1	0.47	108	0.1	0.45	**110**
	0.5	0.53	**63**	2	1.06	**294**	0.3	0.55	**58**	2	0.79	123	0.3	0.83	**26** ^*b*^
Oligomycin	0	0.62	**294**	0	0.96	**435**	0	0.83	**250**	0	0.36	**102**	0	0.57	**204**
	0.5	0.63	**185**	1	1.00	**370**	0.5	0.90	**175**	1	0.46	**41**	0.5	0.53	**123**
	1.0	0.72	**132**	2	0.97	**263**	1	0.93	**119**	2	0.47	**24** ^*b*^	1	0.54	**46**
Vanadate	0	**0.62**	**294**	0	**0.96**	**435**	0	**0.68**	**200**	0	**0.35**	**102**	0	**0.66**	**172**
	0.5	**0.65**	**161**	10	**0.77**	**333**	0.5	**0.61**	**149**	0.2	**0.27**	**65**	0.5	**0.55**	**135**
	2.0	**0.40**	**90**	20	**0.65**	**270**	2	**0.34**	**86**	0.5	**0.20**	**42**	2	**0.40**	**88**

a The data presented are a representative sample of at least two (mostly three or four) independent experiments. The *K_m_* (mM) and *V*_max_ (nmol/min/mg) values that changed appreciably with increasing inhibitor concentrations [I] (μM) are identified; those that decreased are in bold font and those that increased are underlined.

b The kinetic parameters of these Cdr1 variants were less reliable because their ATPase activities in the presence of the indicated inhibitor reached levels that were too close to the background ATPase activity of the sensitive control strain AD/pABC3.

The *K_m_* of the ATPase activity of wt Cdr1 was unaffected by FK506, enniatin B, beauvericin, or oligomycin, but the *V*_max_ values decreased between 41% and 77% with increasing inhibitor concentrations (Table 6). The Lineweaver-Burk plots (Fig. 5A to D, top graphs) indicated noncompetitive inhibition for FK506, enniatin B, beauvericin, and oligomycin, suggesting that the inhibitors did not affect ATP binding at the NBDs of wt Cdr1. Similar patterns (i.e., unchanged *K_m_* and ∼10% to 90% reduced *V*_max_ values) were also observed for the inhibition of the ATPase activities of G521R, M639I, and T1355N by FK506, enniatin B, beauvericin, and oligomycin (Table 6). However, the ATPase activities of A713P responded in a unique and complex manner to the various efflux pump inhibitors. Milbemycin α25, FK506, enniatin B, and beauvericin concentrations that inhibited the ATPase activity of wt Cdr1 by ∼50% did not affect the ATPase activity of A713P at all (Fig. 4A to D), and ∼10-fold higher concentrations of milbemycin α25, enniatin B, and beauvericin actually induced (∼25% to 75%) the ATPase activity of A713P (Fig. 4A, C, and D). The Lineweaver-Burk plots for FK506, enniatin B, and beauvericin action on A713P are presented underneath the plots for wt Cdr1 in Fig. 5A to C. They indicated mixed inhibition (*K_m_* increases and *V*_max_ decreases) for FK506 (Fig. 5A; Table 6), competitive inhibition (*K_m_* increases and *V*_max_ constant) for enniatin B (Fig. 5B; Table 6), and induction (Fig. 5C) (*K_m_* and *V*_max_ increase) (Table 6) of the ATPase activity by beauvericin. These observations were misleading, however, because milbemycin α25, enniatin B, and beauvericin were clearly not inhibitors but actually induced (at ∼10 times the IC_50_ of wt Cdr1) the ATPase activity of A713P. A further increase of the enniatin B and beauvericin concentrations did, however, begin to inhibit the ATPase activity of A713P (see Fig. 4C and D). But contrary to their action on the ATPase activity of A713P, enniatin B, beauvericin, and milbemycin α25 still inhibited FLC efflux of A713P at submicromolar concentrations (Fig. 3B and Table 4). Also, unlike enniatin B and beauvericin which increased the *K_m_* of the A713P ATPase activity more than 2-fold (Table 6), well beyond the *K_m_* of wt Cdr1 (0.62 mM) (Table 5), FK506 and oligomycin increased the *K_m_* of the A713P ATPase activity to 0.74 and 0.47 mM, respectively (Table 6), which were closer to the values for wt Cdr1. Thus, high enniatin B and beauvericin concentrations appeared to reduce the affinity of ATP for the NBD of A713P well beyond that of wt Cdr1, whereas high concentrations of FK506 and oligomycin simply recovered the affinity of ATP in A713P (0.35 mM) (Table 6) to near wild-type levels (i.e., ∼0.62 mM) (Table 6). As expected, vanadate was an uncompetitive inhibitor reducing both the *K_m_* and *V*_max_ values of wt and all four Cdr1 variants (Fig. 5E and Table 6).

The dissociation constants (*K_i_*s) for FK506, enniatin B, beauvericin, oligomycin, and vanadate of the five Cdr1 variants were determined using Dixon plots (Table 7). As expected, they were all comparable but usually somewhat lower than their IC_50_ values (Table 4).

**TABLE 7:** Inhibitor binding constants calculated for the Cdr1 ATPase activities of wt Cdr1 and milbemycin α25-resistant Cdr1 mutants

Strain	*K_i_*^*a*^ (μM) of:				
	FK506	Enniatin B	Beauvericin	Oligomycin	Vanadate
CDR1	1.21 ± 0.24	0.20 ± 0.03	0.08 ± 0.01	0.54 ± 0.01	0.96 ± 0.08
G521R	12.8 ± 4.53	5.34 ± 0.41	2.34 ± 0.20	2.54 ± 0.24	20.8 ± 0.76
M639I	0.56 ± 0.07	0.48 ± 0.04	0.08 ± 0.02	1.15 ± 0.12	1.93 ± 0.13
A713P	11.9 ± 2.8	NA^*b*^	NA	0.43 ± 0.06	0.07 ± 0.01
T1355N	0.29 ± 0.02	0.09 ± 0.06	0.05 ± 0.03	0.22 ± 0.05	2.21 ± 0.46

a The *K_i_* values were calculated using the kinetic results presented in Table 6.

b NA, not applicable because enniatin B and beauvericin induce the ATPase activity of A713P.

### Effects of efflux pump substrates on the ATPase activities of wt and milbemycin α25-resistant Cdr1 mutants.

Some Cdr1 efflux pump substrates are believed to inhibit the ATPase activity by binding to the outward-open conformation of ABC transporters (41, 42). To investigate any possible changes to the affinities of substrates to the outward-open conformation, we measured substrate inhibition of the ATPase activity of wt Cdr1 as well as G521R and A713P.

Most smaller substrates, such as FLC, had no effect on the ATPase activity of Cdr1 (data not shown). Six larger substrates (CLT, KTC, ITC, R6G, MON, and NIG) inhibited the ATPase activity of wt Cdr1; however none inhibited the ATPase activity by more than 75% (see Fig. S5 in the supplemental material). Only CLT, KTC, and R6G inhibited the ATPase activity of wt Cdr1 by more than 50%. The IC_50_ values for wt Cdr1, G521R, and A713P are presented in Table S3 in the supplemental material. The ATPase activity of A713P was more resistant than wt Cdr1 to all six substrates. Its IC_50_ values for CLT (∼55 μM) and KTC (∼100 μM) were 1.8 and 8 times higher, respectively, than those for wt Cdr1, and A713P was resistant to the remaining four substrates (ITC, R6G, MON, and NIG) (Fig. S5). The ATPase activity of G521R was practically resistant to CLT, KTC, MON, and NIG; it was more sensitive to ITC than wt Cdr1; and its R6G susceptibility remained unchanged from wt Cdr1 (Fig. S5). The altered substrate susceptibilities of the ATPase activities of G521R and A713P were independent of whether these compounds were effluxed by these mutant pumps or not. For example, G521R did not efflux CLT and effluxed ITC poorly (Table S2); yet, its ATPase activity was resistant to CLT and more sensitive to ITC than wt Cdr1. Although A713P effluxed MON as efficiently as wt Cdr1 (Table S2) and was unable to efflux NIG, its ATPase activity was insensitive to both compounds (Fig. S5E and F).

### Isolation of enniatin B and beauvericin-resistant Cdr1 efflux pump mutants.

**FIG 6:**
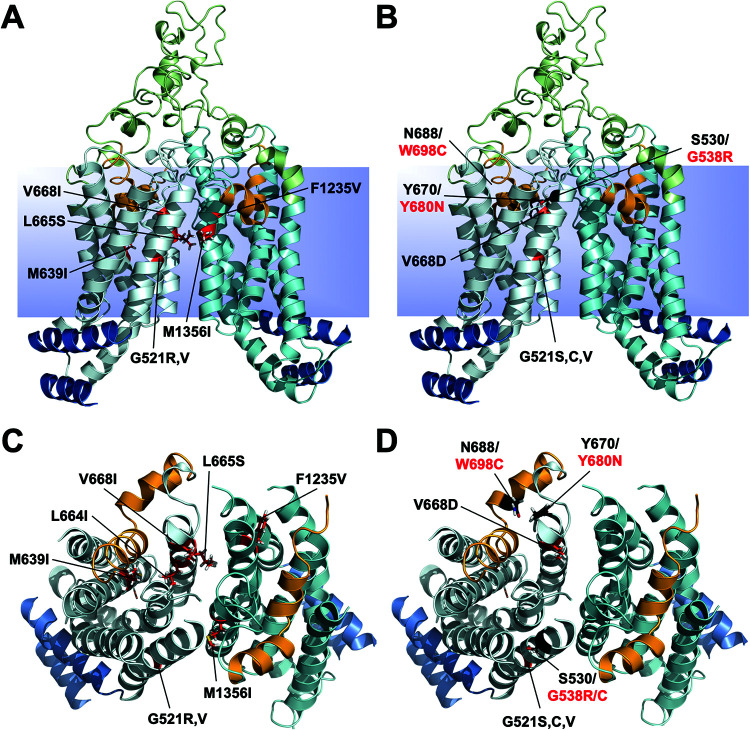
Cartoon models of the TMDs of Cdr1 in the inward-open conformation showing the residues that caused enniatin B and beauvericin resistance. Annotations are as shown in Fig. 1. Residues (red sticks) involved in enniatin B resistance are indicated in A (view from side; L664I is hidden from view) and C (from top), and those involved in beauvericin resistance are shown in B (from side) and D (from top). The mutations are near the center of TMS1 (G521), TMS4 (M639), TMS5 (L664, L665, and V668), TMS8 (V1235), and TMS11 (M1356). The three mutated residues (G538R and G538C, Y680N, and W698C) found in Pdr5 (red letters) caused beauvericin to become a substrate of Pdr5 (43). The equivalent residues (S530, Y670, and N688) in Cdr1 (black letters) are shown as black sticks.

Because the ATPase activities of the four milbemycin α25-resistant mutants responded in a similar fashion to milbemycin α25, enniatin B, and beauvericin, we expected to find Cdr1 efflux pump mutations in similar regions, and possibly identical residues, in a search for enniatin B- and beauvericin-resistant Cdr1 efflux pump mutants. Eight enniatin B- and four beauvericin-resistant efflux pump mutants were isolated (Table 1; see Table S4 and S5 in the supplemental material). The Cdr1 mutations in these variants were indeed similar and some were at identical positions (Fig. 6). As expected, we found Cdr1-G521 mutants in both screens. Two of the eight enniatin B- and three of the four beauvericin-resistant isolates had mutations in G521 (G521R and -V and G521S, -C, and -V, respectively), and M639I was also isolated as an enniatin B-resistant mutant (Fig. 6; Table S4 and S5). The fourth beauvericin-resistant isolate had a mutation (V668D) in the same TMS5 residue as the enniatin B-resistant isolate V668I. The remaining four enniatin B-resistant isolates had mutations in TMS5 (L664I and L665S), in TMS8 (F1235V), and in TMS11 (M1356I) right next to T1355N; the FLC efflux of this mutant was also slightly more resistant to enniatin B but remained susceptible to beauvericin (Fig. 3 and Table 4).

The high frequency of multiple independent milbemycin α25-resistant (75%, 6 of 8 isolates), enniatin B-resistant (25%, 2 of 8 isolates), and beauvericin-resistant (75%, 3 of 4 isolates) isolates with mutations in the same residues (see Table 1) indicated that Cdr1 has rather limited options to develop resistance against these efflux pump inhibitors, especially against beauvericin (i.e., G521S, -C, and -V and V668D) (Fig. 6B and D). Interestingly, despite some significant effort, beauvericin-resistant isolates were found to be mutated only in two positions, both of which were also found to be mutated in a search for enniatin B-resistant isolates (Fig. 6 and Tables S4 and S5). Beauvericin appears to be an almost perfect Cdr1 efflux pump inhibitor. Perhaps this is why nature, through evolution, has selected the production of these depsipeptides by microorganisms as efficient, broad-spectrum, inhibitors of fungal efflux pumps.

## DISCUSSION

There are many unanswered questions concerning PDR transporters, such as how they function, why some compounds are transported 100 or 1,000 times more efficiently than others, what the difference is between a substrate and an inhibitor, and how inhibitors prevent the efflux of pump substrates. What complicates matters further is that more than one substrate can be effluxed simultaneously (e.g., Cdr1) (17) and more than one inhibitor molecule can bind to a substrate binding pocket (e.g., ABCG2) (18). Beauvericin is an example of a broad-spectrum fungal multidrug efflux pump inhibitor that can become an efflux pump substrate under selective pressure on Pdr5 to acquire point mutations at the top of TMS1 (G538R) and TMS5 (Y680N) and in the PDRA motif (12) (W698C) (43), which is in direct contact with Y680N (black residues, Fig. 6B and D). These mutations, however, impaired the transport of typical efflux pump substrates, such as FLC (43). It seems that a multidrug efflux pump can only specialize in the transport of a subset of substrates, and size seems to be a major factor (44, 45). This is probably why fungi have an expanded repertoire of PDR transporters. The Candida krusei multigene family *ABC1*, *ABC11*, and *ABC12*, for example, evolved specialized transport functions optimized for the transport of either smaller (*ABC12*) or larger (*ABC11*) compounds, with *ABC1* having the broadest substrate specificity but apparently at the expense of efflux efficiency of particular compounds (22).

The Cdr1 mutations causing milbemycin α25, enniatin B, and beauvericin resistance were near the center of the TMDs and were mostly different from the previously identified extracellular hot spot 1 and the TMS8-9-10 hot spot 2 regions that enabled cotransport of FK506 with FLC or CHX (17) and were different from the extracellular residues causing RC21v3 resistance (23). Notable exceptions were mutation A713P that was also isolated four times as an FK506-resistant hot spot 1 mutation, and the enniatin B-resistant mutation F1235V in TMS8, which was isolated three times as F1235C, an FK506-resistant hot spot 2 mutation. Milbemycin α25, enniatin B, and beauvericin are true efflux pump inhibitors that possibly bind to the substrate binding pocket of Cdr1 and block the transporter in the inward-open conformation. FK506, however, is a “weak” Cdr1/Pdr5 substrate that interferes with FLC transport (17).

Based on the findings presented in this study, the following models were constructed to explain (i) the ATPase kinetics of wt Cdr1, G521R, and A713P; (ii) how these variants transport substrates; and (iii) how the efflux pump function of wt Cdr1 and the four milbemycin α25-resistant Cdr1 variants are inhibited by various types of efflux pump inhibitors.

### Models for the catalytic cycle of wt Cdr1, G521R, and A713P.

**FIG 7:**
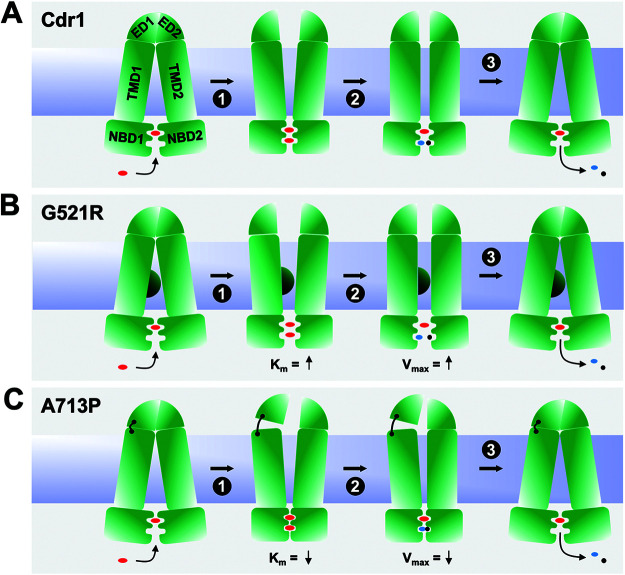
Models for the catalytic cycle of wt Cdr1, G521R, and A713P. The NBDs (NBD1 and NBD2) and TMDs (TMD1 and TMD2) are shown as green rectangles, and the large extracellular domains (ED1 and ED2) are shown as green quadrants. The lipid bilayer is blue, red ovals represent ATP, and the blue ovals and black circles are ADP and phosphate, respectively. The models depict the catalytic cycles of wt Cdr1 (A), G521R (B), and A713P (C). G521R reduces the size of the entry cavity, represented by a semicircle at the center of TMD1, and A713P, represented by a flexible hinge, affects the tight contact between ED1 and TMD1 which is critical for the constitutively high basal ATPase activity of Cdr1. The increased (G521R) or decreased (A713P) *K_m_* and *V*_max_ values are due to larger or reduced distances, respectively, between NBD1 and NBD2, which caused lower (B) or higher (C) affinities of ATP and vanadate for CNBD2.

Models that account for the kinetic properties of the ATPase activity of wt Cdr1, G521R, and A713P are presented in Fig. 7. There is evidence that ATP may bind at the noncanonical composite NBD1 (CNBD1) of Cdr1 at all times (42) but is not hydrolyzed (46), although Furman et al. proposed nucleotide exchange at CNBD1 as an integral part of the Pdr5 transport cycle (47). In Fig. 7A (left panel) wt Cdr1 is in the inward-open conformation with one ATP molecule bound to CNBD1. In step 1 of the transport cycle, a second ATP molecule binds to the catalytically active CNBD2, which causes a rigid body motion between the two NBDs that triggers large conformational changes at the TMDs and switches the transporter into the outward-open conformation. To revert the transporter back to the inward-open conformation, one ATP is hydrolyzed at CNBD2 causing Cdr1 to assume an intermediate conformation (step 2), and the release of ADP and phosphate Pi completes the transport cycle (step 3). Increasing the size of residue 521 (G521R, Fig. 7B) affects a critical contact point between the two TMDs which has dramatic consequences—it affects the cross talk between the NBDs and the TMDs resulting in increased *K_m_* (step 1), increased *V*_max_ (step 2), and an ∼20-fold increased vanadate dissociation constant *K_i_* (Table 5 and 7). The model accounts for these changes in *K*_m_ and *V*_max_—the distance between the two NBDs in G521R is greater in step 1 and step 2 than for wt Cdr1 (Fig. 7B). A713 is a critical EL1-EL3 contact residue (Fig. 7C, black hinge between TMD1 and ED1) such that A713P causes FK506 (17) and milbemycin α25, but not enniatin B and beauvericin, to no longer inhibit FLC efflux (Fig. 3). Loosening this contact in A713P impairs proper opening of the EDs (step 1), which affects ATP binding (decreased *K_m_* in step 1 and decreased *V*_max_ in step 2) (Table 5) and ATP hydrolysis (∼14 times reduced *K_i_* for vanadate) (Table 7) at CNBD2. The reduced TMD1-ED1 contact forces the NBDs closer together (steps 2 and 3) and, thus, increases the affinities for ATP and vanadate and reduces its ATPase activity and turnover rate, *k*_cat_, by ∼70% (Table 5; Fig. 7C). Thus, the increased (G521R) or decreased (A713P) *K_m_* and *V*_max_ values are due to increased or reduced distances between NBD1 and NBD2, which causes lower (Fig. 7B) or higher (Fig. 7C) affinities of ATP and vanadate for CNBD2.

### Models for Cdr1 substrate transport.

**FIG 8:**
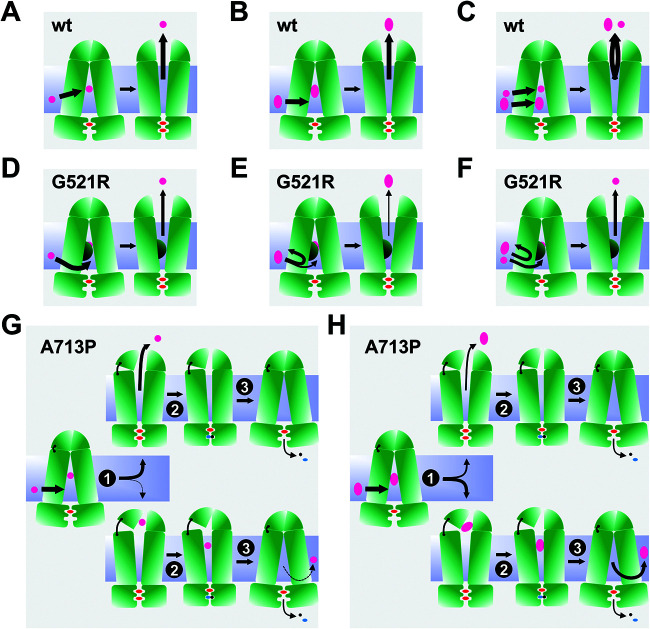
Substrate transport models for wt Cdr1, G521R, and A713P. Arrows indicate entry or exit of substrate, and U-turns mean the substrate cannot enter the transporter. The thickness of the arrows indicates how efficiently a substrate can be transported relative to wt Cdr1. Models for the transport of small substrates (magenta circles) and large substrates (magenta ovals) and the cotransport of two substrates by wt Cdr1 are shown in A, B, and C, respectively. Models D, E, and F show substrate transport by G521R. G521R can also transport small substrates, although at slightly reduced efficiencies (D), but it is severely affected or even unable to transport larger substrates because their access through the smaller entry gate is restricted (E). Models in G and H show how the relaxed TMD1-ED1 contact region in Cdr1-A713P affects the transport of small substrates (G) less than the transport of larger substrates (H). While all small substrates are transported unhindered, a proportion of the larger substrates remains stuck inside the transporter and only a small fraction is transported. The top rows in G and H show the fate of the transported substrate, and the bottom rows show the fate of the substrate that is not transported.

Models accounting for the transport properties of wt Cdr1, G521R, and A713P are presented in Fig. 8. Wt Cdr1 can transport small (Fig. 8A) and larger (Fig. 8B) substrates, and it can even transport two substrates at the same time (17) (Fig. 8C) as long as their binding sites do not overlap. The substrate specificity of G521R was dramatically different; it preferentially transported smaller substrates and was unable to efficiently transport large compounds, such as PSC (Fig. 2). We propose that G521 is a gatekeeper, which determines whether substrates and inhibitors can enter the transporter through an opening between TMS1 and TMS11 (Fig. 1C and D and Fig. 6). The smaller gate in R521 impairs entry, and hence efflux, of larger substrates more than smaller substrates, as indicated by the thinner efflux arrows in Fig. 8D and E than those in Fig. 8A and B. The proposed size selection function for G521R is further supported by findings of Kolaczkowski and colleagues who identified G521S and G521D as mutations in Cdr1 that enabled resazurine and resorufine, two small Snq2 substrates, to also become substrates of Cdr1 (48). The bottom two models in Fig. 8 explain how the efflux of some larger compounds is more severely impaired in A713P (Fig. 8H) than the efflux of smaller compounds (Fig. 8G). A713P also had a size-dependent efflux pump phenotype with 16- to 32-fold reduced efflux for PSC, ITC, and NIG (MW of >700 Da), whereas the efflux of small- and medium-sized compounds was reduced only ∼2- to 4-fold relative to wt Cdr1. The only exceptions were CLT (MW of 345 Da), MCZ (MW of 416 Da), and KTC (MW of 531 Da), the transport efficiencies of which were 8-fold lower in A713P (Table 2). However, unlike for G521R, the reduced transport efficiencies of A713P for larger substrates were not caused by an impaired access to the substrate binding pocket because the inhibition of FLC efflux by enniatin B and beauvericin (Fig. 3B) and the inhibition of the ATPase activity by oligomycin (Fig. 4E), three of the largest test compounds, were unchanged in A713P. This finding would suggest that a large proportion of CLT, MCZ, KTC, PSC, ITC, and NIG simply got stuck inside the transporter but this did not prevent the opening and closing of the transporter because the ATPase activity of A713P was much less severely inhibited by any of these compounds (Fig. S5). For a lack of a better term, such compounds could be considered nontransported substrates of A713P. A small fraction of some of these larger substrates, however, was still effluxed by A713P even though the majority was possibly released into the lipid bilayer after completion of the transport cycle (Fig. 8H).

### Models for the inhibition of wt Cdr1, G521R, and A713P efflux pumps.

**FIG 9:**
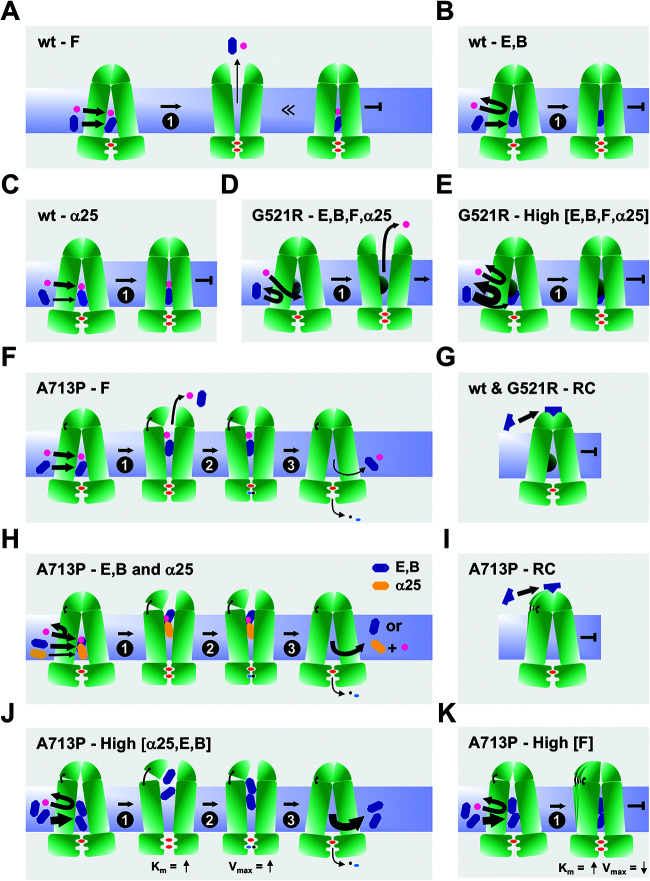
Models for efflux pump inhibition of wt Cdr1, G521R, and A713P. Arrows of various thickness indicate the direction and magnitude of the flow of substrate (FLC) and/or inhibitors. Thinner or thicker arrows indicate that significantly less or more inhibitor is required to inhibit the FLC efflux pump function than the ATPase activity. The substrate is FLC (magenta circle). Inhibitors FK506 (F), milbemycin α25 (α25), enniatin B (E), and beauvericin (B) are represented by blue hexagons and RC21v3 (RC) is represented by blue traffic cones. Models in A to C show transport inhibition of FLC by the four efflux pump inhibitors FK506, milbemycin α25, enniatin B, and beauvericin in wt Cdr1. (A) A small fraction of FK506 is cotransported with FLC (center), but the majority of FK506 molecules block the transporter in the inward-open conformation (right). (B) “True” inhibition by enniatin B and beauvericin blocking the transporter in the inward-open conformation. (C) “Cooperative” inhibition by the milbemycin α25-FLC combination. Models in D and E show transport inhibition in G521R. (D) The smaller gate in G521R prevents access by inhibitors. (E) Much higher inhibitor concentrations are required to inhibit FLC efflux. Models in G and I show the interactions of RC21v3 with transporters. (G) RC21v3 binds to the extracellular domain of the inward-open conformation of Cdr1 and G521R with similar affinities. (I) The hinge destabilizes this region which leads to a slightly reduced RC21v3 affinity in A713P. Models in F, H, J, and K show transport inhibition in A713P. (F) A larger portion of FK506 (blue hexagon) than in wt Cdr1 is cotransported with FLC, but unlike in wt Cdr1, the remaining portion stuck inside the transporter does not block the completion of the transport cycle. (H) Milbemycin α25 (orange hexagon) and FLC and enniatin B and beauvericin (blue hexagon) inhibit the FLC efflux of A713P as for wt Cdr1, although they do not block the conformational change of the transporter nor inhibit the ATPase activity. In J and K, the hinge allows two molecules of milbemycin α25, enniatin B, beauvericin, and FK506 to enter the transporter, thereby separating the two NBDs further and increasing the *K_m_* for ATP. (J) High concentrations of milbemycin α25, enniatin B, and beauvericin (i.e., two molecules) induce the ATPase activity of A713P. In K, however, high concentrations of FK506 (i.e., two molecules) block the transporter in the inward-open conformation, thus inhibiting FLC transport and the ATPase activity of A713P.

Models for the interactions of efflux pump inhibitors with wt Cdr1, G521R, and A713P are presented in Fig. 9. FK506 is an efflux pump inhibitor but, unlike enniatin B and beauvericin, a small fraction of FK506 can also be transported by wt Cdr1 (Fig. 9A). The model in Fig. 9A shows how FK506, a weak substrate, interferes with FLC transport, as only a small proportion of FK506 and FLC are actually cotransported (center)—the rest of FK506 and FLC block the transporter in the inward-open conformation (right) like enniatin B and beauvericin (Fig. 9B). Enniatin B and beauvericin inhibit Cdr1 efflux pump function by blocking the transporter in the inward-open conformation. It is unclear whether FLC can still enter the transporter in the presence of these inhibitors or whether enniatin B and beauvericin prevent entry of FLC, as presented in Fig. 9B. Milbemycin α25 is an inhibitor for which binding to the substrate binding pocket of wt Cdr1 was enhanced by the presence of FLC possibly binding nearby so that together they inhibited wt Cdr1 (and also M639I, and to a lesser degree T1355N and G521R) (Table 4) by blocking the transporter in the inward-open conformation (Fig. 9C).

At concentrations that inhibited wt Cdr1, the access of inhibitors to the substrate binding pocket of G521R was prevented and FLC efflux occurred unobstructed (Fig. 9D). Much higher inhibitor concentrations were required to inhibit FLC efflux because of the restriction of large compounds through the entry gate (Fig. 9E). The fact that milbemycin α25 inhibition of FLC efflux was still partially (3.5-fold) enhanced by FLC (Table 4) indicates that some FLC was still able to enter G521R even at high milbemycin α25 concentrations (this particular case is not shown in Fig. 9D).

Models explaining the complex interactions of the efflux pump inhibitors with A713P are presented in Fig. 9F and H to J. Milbemycin α25, enniatin B, and beauvericin induced the ATPase activity of A713P (Fig. 4A, C, and D), even though its FLC efflux pump function was as sensitive to enniatin B as wt Cdr1 and only 2 and 5 times more resistant than wt Cdr1 to beauvericin and milbemycin α25, respectively (Fig. 3B, Table 4). A simple explanation for this result would be that these inhibitors had become substrates of A713P that competitively inhibited FLC efflux. This explanation, however, was clearly not the case because the MICs of enniatin B and beauvericin for cells overexpressing A713P were the same as that for the sensitive control strain AD/pABC3 (see Fig. S6A and B in the supplemental material). Enniatin B and beauvericin possibly inhibit FLC efflux by binding to the substrate binding pocket of A713P like in wt Cdr1 (Fig. 9B), but instead of locking the transporter, the flexible TMD1-ED1 contact of A713P enables completion of the transport cycle (Fig. 9H), which is similar to the futile transport of large substrates by A713P (bottom panel in Fig. 8H). The situation is slightly different for milbemycin α25 (orange hexagon in Fig. 9H). In the presence of milbemycin α25, FLC still enters A713P as with wt Cdr1, but unlike freezing wt Cdr1 in the open conformation, milbemycin α25 and FLC do not freeze A713P in the open conformation but remain stuck inside and are possibly released through the entry gate after completion of the transport cycle, which is similar to results with enniatin B and beauvericin (Fig. 9H). The milbemycin α25 and FLC binding sites (Fig. 9H) remain much like those in wt Cdr1 (Fig. 9C) because FLC enhances milbemycin α25 binding to A713P even more (∼75 times) effectively than to wt Cdr1 (∼35 times) (Table 4).

At higher concentrations, FK506 only partially inhibited the ATPase activity, and milbemycin α25, enniatin B, and beauvericin even induced the ATPase activity of A713P (Fig. 4A to D). The models in Fig. 9J and K account for these observations. We propose that at high inhibitor concentrations, two inhibitor molecules enter A713P which affects the TMD-NBD interaction and partially restores the impaired *K_m_* (0.35 mM) and *V*_max_ (101 nmol/min/mg) values of the A713P ATPase activity. Two enniatin B and beauvericin molecules separate the NBDs further than in wt Cdr1, thus increasing the *K_m_* to 0.81 mM and 0.79 mM (Table 6), respectively. The flexible TMD1-ED1 contact of A713P enables completion of the transport cycle even in the presence of two milbemycin α25, enniatin B, or beauvericin molecules (Fig. 9J). We propose the pump function to be different for FK506 where possibly two FK506 molecules, at ∼20 times the IC_50_ value for wt Cdr1, recover the impaired *K_m_* (0.35 mM) value of the A713P ATPase activity but, unlike with the other inhibitors, two FK506 molecules partially (∼25%) (Fig. 4B and Table 6) inhibit the ATPase activity of A713P (Fig. 9K). Interestingly, increasing the enniatin B or beauvericin concentrations even further (>8 μM) began to reduce the A713P ATPase activity (Fig. 4C and D). Perhaps three enniatin B or beauvericin molecules are required in the substrate binding pocket to inhibit the ATPase activity of A713P by locking the transporter in the inward-open conformation (Fig. 9K).

RC21v3 binds with similar affinities to the extracellular domain in the inward-open conformation of wt Cdr1 and G521R (Fig. 9G). The dramatically reduced substrate binding affinity to the outward-open conformation of G521R and A713P (Fig. S5), but their only marginally increased RC21v3 resistance (Fig. 3A), supports this model. The model in Fig. 9I shows how the slightly increased RC21v3 resistance of A713P (Fig. 3A) could be caused by subtle changes to the extracellular binding site due to the impaired TMD1-ED1 contact.

The oligomycin sensitivities of the ATPase activities were least affected by any of the Cdr1 mutations (Fig. 4E), suggesting that oligomycin is a noncompetitive inhibitor of all Cdr1 variants, even A713P. As expected, the ATPase activity of G512R was ∼3 times more resistant to oligomycin, a large compound.

### Efflux pump inhibition of M639I and T1355N.

M639I and T1355N mutations caused only subtle changes to the substrate transport efficiencies and the inhibitor susceptibilities of wt Cdr1. T1355 was previously noted as an important residue involved in substrate transport and inhibitor binding, both in Cdr1 (49) and Pdr5 (50, 51). T1355A and T1355S were unaffected in FLC and R6G transport, but T1355F could not transport either substrate and only T1355A was resistant to the FK506 analogue FK520 (49). The Cdr1-T1355 equivalent mutations in Pdr5, namely, T1364S, T1364A, and T1364F, were partially (T1364S) or almost completely (T1364A and T1364F) resistant to FK506, but only T1364S and T1364A were able to efflux FLC (50, 51). We isolated Pdr5-T1364S in our previous screen for FK506-resistant FLC efflux pump mutants, but no FK506 resistant mutants were isolated for the equivalent residue in Cdr1-T1355 (17). In the current screen, T1355N was isolated as a milbemycin α25-resistant mutant that was cross-resistant to enniatin B, and we isolated M1356I as an enniatin B-resistant mutant. It would seem that M639, giving rise to both milbemycin α25- and enniatin B-resistant mutants, and T1355 are important residues involved in substrate transport and efflux pump inhibition of Cdr1. These residues are likely to be important TMS-contact points during opening and closing of the transporter. Alternatively, T1355, which faces the substrate binding pocket of Cdr1, could contribute to the binding of the substrate and/or inhibitor (Fig. 1C and D and Fig. 6A and C). Subtle changes in these contact residues may affect the interaction of substrate with inhibitor in the substrate binding pocket and favor FLC efflux over inhibitor binding, possibly by steric interference. Although M639I and T1355N remained sensitive to all five Cdr1 efflux pump inhibitors, ∼5 times higher milbemycin α25 and ∼2 to 4 times higher enniatin B concentrations (the inhibitors used for mutant selection) were required to inhibit FLC efflux (Table 4 and Fig. 3B).

### Model for the Cdr1 transport function.

Recently, the first PDR transporter structures, namely, Pdr5 in the open and closed conformation, were published (52). The structures are in complete agreement with the models described above. The authors identified two novel linker domains (LD1 and LD2) as characteristic features of PDR transporters. They comprise helix H3 near the N terminus and H17 just after TMS6, each with an attached loop region, that are in direct contact with the noncanonical CNBD1. We previously reported the highly conserved H17 motif as a conserved asymmetric feature of PDR transporters, although it was predicted as a conserved beta-sheet just after TMS6 (12). An interesting consequence of this novel “asymmetric” motif is that Pdr5 has only one opening, between TMS1 and TMS11, as the second pseudosymmetric entrance cavity between TMS5 and TMS7 (Fig. 1C) was found to be closed in both conformations (52). This finding strengthens the proposed gate-keeper function for Cdr1-G521 at the center of TMS1 (Fig. 1C). The reported structures also confirmed the three predicted disulfide bonds between C712 and C732 (1), C1418 and C1441 (2), and C1402 and C1444 (3) for our *ab initio* model of the extracellular domain of Cdr1 (17). The tight contact between the FK506-resistant Pdr5/Cdr1 hot spot 1 residues A723/A713 of EL3 with EL1 (17), which correlates to the proposed flexible hinge region for A713P in Fig. 7 and 9, was also confirmed. Although these structures represent a significant milestone in PDR transporter structure and function elucidation, there are valid concerns as to how accurately these structures reflect Pdr5 in native plasma membranes (53). The detergent-extracted Pdr5 particles that were reconstituted into peptidiscs had an ∼96% lower ATPase activity (73 nmol/min/mg) than Pdr5 (∼2,000 nmol/min/mg) in native plasma membranes (46) possibly due to some constriction of the protein by the peptidisc surrounding the transporter (52). Yeast plasma membranes are highly organized structures with a number of distinct subcompartments (54) of defined protein and lipid composition that provide a flexible environment for conformational changes of membrane proteins (55). This is why it is so important to scrutinize membrane protein structures with additional experimental evidence (53).

### Peristaltic pump model for PDR transporters.

Based on the current literature and the results presented in this report, we propose the following model for PDR transporters. PDR transporters are constitutively active asymmetric ABC transporters. Their characteristic LDs connect TMD1 with NBD2 (52) so that “gate 2” between TMS5 and TMS7 (Fig. 1C) is closed and the noncanonical CNBD1 directly underneath binds, but does not hydrolyze, ATP at all times (52). The EDs with their characteristically large EL3 and EL6 are tightly linked to the TMDs and the NBDs so that the canonical CNBD2 underneath the entrance cavity between TMS1 and TMS11 cannot be activated via an induced fit-type mechanism which is the hallmark of ABCB type transporters (56) lacking such large EDs. ATP binding at CNBD2 induces the closure of gate 1 between TMS1 and TMS11 and triggers large conformational changes that forces efflux pump substrates through a hydrophobic plug (15, 52) into the extracellular space. The hydrophobic plug probably behaves like an “elastic band” that adjusts to the size of the efflux pump substrate being squeezed through this hydrophobic plug. This elasticity possibly determines how efficiently substrates can be transported. If it is too loose or too tight, substrates cannot be transported effectively; smaller substrates are less efficiently transported or not transported at all, optimum-sized substrates are most efficiently transported (Fig. 2), and compounds that are too large or conformationally constrained (most efflux pump inhibitors are cyclic compounds) become inhibitors. The Pdr5 TMS1, TMS5, and PDRA mutations that enabled efficient beauvericin transport (Fig. 6B and D) (43) and the Pdr5/Cdr1 hot spot 1 and hot spot 2 mutations (17) which improved FK506 transport possibly increased the elasticity of this elastic band which negatively impacted the efflux efficiency of smaller substrates. Naturally, such conformational flexibility must be influenced strongly by the interactions of PDR transporters with their native lipid environment (55).

Confirmation of these models will come from atomic-resolution structures for the protein embedded in native lipids with and without substrates and inhibitors present, which is the focus of our current work.

In conclusion, there appears to be only one way for FLC transport to become resistant to inhibition by noncompetitive Cdr1 efflux pump inhibitors, such as milbemycin α25, enniatin B, and beauvericin, and that is to prevent entry (i.e., by mutating G521) of these typically large, circular compounds. Two options are available, however, for FLC transport to become resistant to inhibition by weak substrates, such as FK506. Option 1 is to prevent entry (G521 mutations), and option 2 is to enhance cotransport (i.e., via hot spot 1 and 2 mutations) of FLC with FK506. A detailed characterization of the milbemycin α25-, enniatin B-, and beauvericin-resistant mutants provided important clues about how efflux pump substrates and inhibitors interact with Cdr1. We discovered a gate function for G521 and propose that A713 contributes to a critical contact point at the top of the transporter involved in the constitutively high, basal, noninducible ATPase activity of Cdr1. Interestingly, the action of some inhibitors was influenced by the presence of FLC in the substrate binding cavity of Cdr1. Some inhibitor/FLC combinations (i.e., milbemycin α25/FLC in all Cdr1 variants) enhanced, while others (i.e., enniatin B/FLC and beauvericin/FLC in T1355N) reduced, the inhibitory potential of the broad-spectrum efflux pump inhibitors. The rather complex and combinatorial interactions have important implications for the design and development of novel efflux pump inhibitors. Our results indicate that an ideal efflux pump inhibitor should not be an efflux pump substrate; rather, it should have broad-spectrum activity against a range of efflux pumps, and it should not be compromised by the presence of other antifungal efflux pump substrates.

## MATERIALS AND METHODS

### Materials.

Milbemycin α25 was a generous gift from Daiichi Sankyo Co., Ltd. (Tokyo, Japan). FK506 was kindly provided by Astellas Pharma Inc. (Tokyo, Japan). FLC was purchased from LKT Laboratories Inc. (St. Paul, MN). Clotrimazole (CLT), miconazole (MCZ), ketoconazole (KTC), itraconazole (ITC), voriconazole (VRC), posaconazole (PSC), nigericin (NIG), monensin (MON), latrunculin A (LaA), cerulenin (CER), rhodamine 6G (R6G), beauvericin, and amphotericin B (AMB) were obtained from Sigma-Aldrich Japan Inc. (Tokyo, Japan). Enniatin B was purchased from Alexis Biochemicals (San Diego, CA). Cycloheximide (CHX) was purchased from Nacalai Tesque Inc. (Kyoto, Japan). RC21v3 was our in-house-developed d-octapeptide *Candia albicans* Cdr1-specific inhibitor (23).

### Yeast strains and culture conditions.

S. cerevisiae strains used in this study are listed in Table 1. The S. cerevisiae strain used to overexpress Cdr1 is based on AD1-8u^-^ (34). Strains AD/pABC3 and AD/CDR1 were constructed by integrating transformation cassettes from plasmids pABC3 and pABC3-CDR1B (CDR1 B allele of C. albicans 10261) (57) at the *PDR5* locus of AD1-8u^-^ as described previously (20). Yeast cells were cultured routinely in 1% (wt/vol) yeast extract, 2% (wt/vol) peptone, and 2% (wt/vol) glucose (YPD) medium (Difco Laboratories, Detroit, MI) at 30°C. Cells used to determine the MIC of antifungals were grown to mid-log phase in buffered complete supplement mixture without uracil (CSM-URA) medium (pH 7.0) (58). Strain AD1-8u^-^ was grown in buffered CSM-URA medium supplemented with 0.02% (wt/vol) uridine.

### Determination of the MIC of antifungals.

The susceptibility of yeast to antifungal agents was determined using a modification of the CLSI M27-A3 broth microdilution reference method (59) as described previously (60). This modification was necessary because AD1-8u^-^ and its derivative strains do not grow in RPMI medium used in the CLSI method. The MIC for an antifungal was defined as the lowest concentration of the drug that inhibited growth yield by at least 90%.

### Agarose diffusion chemosensitization assay.

Compounds were tested for inhibition of FLC transport by yeast strains overexpressing Cdr1 or Cdr1 mutations. Petri dishes containing FLC at 0.25× MIC in solidified YPD were seeded with 1 × 10^6^ mid-logarithmic-phase yeast cells suspended in 5 mL of melted (50°C) top-agarose medium (YPD plus 0.6% [wt/vol] agarose supplemented with FLC at 0.25× MIC). Filter discs containing 5 μg milbemycin α25, 10 μg FK506, 0.2 μg enniatin B, or 0.5 μg beauvericin were placed onto the solidified top agarose, and the plates were incubated at 30°C for 48 h.

### Checkerboard chemosensitization assay.

This two-dimensional liquid medium microtiter plate assay measures the MIC_FLC_ at various concentrations of a test compound in order to identify potential synergistic effects between the test compound and FLC. The assay was carried out in CSM medium (pH 7.0) as described previously (58). FICI values are quantitative measures for combination therapies; values of ≤0.5 indicate “synergy,” values of >0.5 to 4 indicate “no interaction,” and values of >4 indicate “antagonism” between two drugs (61, 62).

### Isolation of Cdr1 inhibitor-resistant FLC efflux pump mutants.

A lawn of 1 × 10^5^ AD/CDR1 cells was plated on YPD agar containing 0.25× MIC of FLC and 5 μg of milbemycin α25, 0.2 μg of enniatin B, or 0.5 μg of beauvericin per disc. Inhibitor-resistant colonies of AD/CDR1 from within growth inhibitory zones on the YPD agar were picked. Genomic DNA was extracted from individual inhibitor-resistant colonies, and the entire PDR5::CDR1-URA3 transformation cassette was amplified by PCR. The *CDR1* ORF of each suppressor mutant transformation cassette was sequenced to identify any mutations present. The cassette was then used to transform host strain S. cerevisiae AD1-8u^-^ to confirm that mutations identified in *CDR1* conferred inhibitor resistance (23).

### Isolation of plasma membrane fractions and measurement of *in vitro* Cdr1-ATPase activity.

Yeast cells were grown in 40 mL YPD medium at 30°C to mid-exponential phase (optical density at 600 nm [OD_600_], 3), and small-scale plasma membrane preparations were used to determine the kinetic properties of the ATPase activities of Cdr1 variants. Harvesting 40 optical density units (ODU; 1 ODU = 1-mL cell culture of an OD_600_ of 1) of cells at mid-exponential growth phase and using a small-scale plasma membrane isolation protocol optimized for 40 ODU of cells (63, 64) gave ∼3 times higher Cdr1 ATPase activities than the large-scale (1,750 ODU of cells) plasma membrane isolation protocol which uses 250-mL YPD cell cultures harvested at diauxic phase (OD_600_, 7) (20, 58). Large-scale plasma membrane preparations were used to determine expression levels and substrate and inhibitor susceptibilities of the Cdr1 ATPase activities presented in Fig. 1 and 4, Fig. S3, Table 3, and Table S3. The small-scale plasma membrane isolation protocol, however, was used to determine the kinetic properties of the ATPase activities of the Cdr1 variants. Protein content was determined using a micro-Bradford protein assay kit II (Bio-Rad, Hercules, CA). The ATPase activities of individual plasma membrane preparations were measured as described previously (58) The kinetic parameters for the Cdr1 ATPase activities in the presence and absence of an inhibitor were determined with Lineweaver-Burk plots, and the *K_i_* values were determined using the Dixon plot.

### Data availability.

All data are contained within the manuscript and the supplemental information.

## References

[1] El-Awady R, Saleh E, Hashim A, Soliman N, Dallah A, Elrasheed A, Elakraa G. 2016. The role of eukaryotic and prokaryotic ABC transporter family in failure of chemotherapy. Front Pharmacol 7:535. 10.3389/fphar.2016.00535. 28119610 PMC5223437

[2] Cannon RD, Lamping E, Holmes AR, Niimi K, Baret PV, Keniya MV, Tanabe K, Niimi M, Goffeau A, Monk BC. 2009. Efflux-mediated antifungal drug resistance. Clin Microbiol Rev 22:291–321. 10.1128/CMR.00051-08. 19366916 PMC2668233

[3] Dassa E, Bouige P. 2001. The ABC of ABCS: a phylogenetic and functional classification of ABC systems in living organisms. Res Microbiol 152:211–229. 10.1016/s0923-2508(01)01194-9. 11421270

[4] Holland IB, Blight MA. 1999. ABC-ATPases, adaptable energy generators fuelling transmembrane movement of a variety of molecules in organisms from bacteria to humans. J Mol Biol 293:381–399. 10.1006/jmbi.1999.2993. 10529352

[5] Dean M, Allikmets R. 2001. Complete characterization of the human ABC gene family. J Bioenerg Biomembr 33:475–479. 10.1023/a:1012823120935. 11804189

[6] Dean M, Rzhetsky A, Allikmets R. 2001. The human ATP-binding cassette (ABC) transporter superfamily. Genome Res 11:1156–1166. 10.1101/gr.184901. 11435397

[7] Kang J, Park J, Choi H, Burla B, Kretzschmar T, Lee Y, Martinoia E. 2011. Plant ABC transporters. Arabidopsis Book 9:e0153. 10.1199/tab.0153. 22303277 PMC3268509

[8] Kovalchuk A, Driessen AJ. 2010. Phylogenetic analysis of fungal ABC transporters. BMC Genomics 11:177. 10.1186/1471-2164-11-177. 20233411 PMC2848647

[9] Panapruksachat S, Iwatani S, Oura T, Vanittanakom N, Chindamporn A, Niimi K, Niimi M, Lamping E, Cannon RD, Kajiwara S. 2016. Identification and functional characterization of *Penicillium marneffei* pleiotropic drug resistance transporters *ABC1* and *ABC2*. Med Mycol 54:478–491. 10.1093/mmy/myv117. 26782644

[10] Paumi CM, Chuk M, Snider J, Stagljar I, Michaelis S. 2009. ABC transporters in *Saccharomyces cerevisiae* and their interactors: new technology advances the biology of the ABCC (MRP) subfamily. Microbiol Mol Biol Rev 73:577–593. 10.1128/MMBR.00020-09. 19946134 PMC2786581

[11] Watanasrisin W, Iwatani S, Oura T, Tomita Y, Ikushima S, Chindamporn A, Niimi M, Niimi K, Lamping E, Cannon RD, Kajiwara S. 2016. Identification and characterization of *Candida utilis* multidrug efflux transporter CuCdr1p. FEMS Yeast Res 16:fow042. 10.1093/femsyr/fow042. 27188883

[12] Lamping E, Baret PV, Holmes AR, Monk BC, Goffeau A, Cannon RD. 2010. Fungal PDR transporters: phylogeny, topology, motifs and function. Fungal Genet Biol 47:127–142. 10.1016/j.fgb.2009.10.007. 19857594 PMC2814995

[13] Kim Y, Chen J. 2018. Molecular structure of human P-glycoprotein in the ATP-bound, outward-facing conformation. Science 359:915–919. 10.1126/science.aar7389. 29371429

[14] Manolaridis I, Jackson SM, Taylor NMI, Kowal J, Stahlberg H, Locher KP. 2018. Cryo-EM structures of a human ABCG2 mutant trapped in ATP-bound and substrate-bound states. Nature 563:426–430. 10.1038/s41586-018-0680-3. 30405239 PMC6379061

[15] Taylor NMI, Manolaridis I, Jackson SM, Kowal J, Stahlberg H, Locher KP. 2017. Structure of the human multidrug transporter ABCG2. Nature 546:504–509. 10.1038/nature22345. 28554189

[16] Lee JY, Kinch LN, Borek DM, Wang J, Wang J, Urbatsch IL, Xie XS, Grishin NV, Cohen JC, Otwinowski Z, Hobbs HH, Rosenbaum DM. 2016. Crystal structure of the human sterol transporter ABCG5/ABCG8. Nature 533:561–564. 10.1038/nature17666. 27144356 PMC4964963

[17] Tanabe K, Bonus M, Tomiyama S, Miyoshi K, Nagi M, Niimi K, Chindamporn A, Gohlke H, Schmitt L, Cannon RD, Niimi M, Lamping E. 2019. FK506 resistance of *Saccharomyces cerevisiae* Pdr5 and *Candida albicans* Cdr1 involves mutations in the transmembrane domains and extracellular loops. Antimicrob Agents Chemother 63:e01146-18. 10.1128/AAC.01146-18. 30348662 PMC6325234

[18] Jackson SM, Manolaridis I, Kowal J, Zechner M, Taylor NMI, Bause M, Bauer S, Bartholomaeus R, Bernhardt G, Koenig B, Buschauer A, Stahlberg H, Altmann KH, Locher KP. 2018. Structural basis of small-molecule inhibition of human multidrug transporter ABCG2. Nat Struct Mol Biol 25:333–340. 10.1038/s41594-018-0049-1. 29610494

[19] James JE, Lamping E, Santhanam J, Cannon RD. 2021. PDR transporter *ABC1* Is involved in the innate azole resistance of the human fungal pathogen *Fusarium keratoplasticum*. Front Microbiol 12:673206. 10.3389/fmicb.2021.673206. 34149660 PMC8211738

[20] Lamping E, Monk BC, Niimi K, Holmes AR, Tsao S, Tanabe K, Niimi M, Uehara Y, Cannon RD. 2007. Characterization of three classes of membrane proteins involved in fungal azole resistance by functional hyperexpression in *Saccharomyces cerevisiae*. Eukaryot Cell 6:1150–1165. 10.1128/EC.00091-07. 17513564 PMC1951111

[21] Lamping E, Ranchod A, Nakamura K, Tyndall JD, Niimi K, Holmes AR, Niimi M, Cannon RD. 2009. Abc1p is a multidrug efflux transporter that tips the balance in favor of innate azole resistance in *Candida krusei*. Antimicrob Agents Chemother 53:354–369. 10.1128/AAC.01095-08. 19015352 PMC2630665

[22] Lamping E, Zhu JY, Niimi M, Cannon RD. 2017. Role of ectopic gene conversion in the evolution of a *Candida krusei* pleiotropic drug resistance transporter family. Genetics 205:1619–1639. 10.1534/genetics.116.194811. 28159755 PMC5378117

[23] Niimi K, Harding DR, Holmes AR, Lamping E, Niimi M, Tyndall JD, Cannon RD, Monk BC. 2012. Specific interactions between the *Candida albicans* ABC transporter Cdr1p ectodomain and a D-octapeptide derivative inhibitor. Mol Microbiol 85:747–767. 10.1111/j.1365-2958.2012.08140.x. 22788839 PMC3418399

[24] Monk BC, Keniya M. 2017. Using yeast to discover inhibitors of multidrug efflux in *Candida albicans*, p 491–544. *In* Prasad R (ed), *Candida albicans*: cellular and molecular biology, 2nd ed. Springer International, New York, NY.

[25] Mealey KL, Bentjen SA, Gay JM, Cantor GH. 2001. Ivermectin sensitivity in collies is associated with a deletion mutation of the mdr1 gene. Pharmacogenetics 11:727–733. 10.1097/00008571-200111000-00012. 11692082

[26] Wolstenholme AJ, Rogers AT. 2005. Glutamate-gated chloride channels and the mode of action of the avermectin/milbemycin anthelmintics. Parasitology 131:S85–S95. 10.1017/S0031182005008218. 16569295

[27] Holmes AR, Lin YH, Niimi K, Lamping E, Keniya M, Niimi M, Tanabe K, Monk BC, Cannon RD. 2008. ABC transporter Cdr1p contributes more than Cdr2p does to fluconazole efflux in fluconazole-resistant *Candida albicans* clinical isolates. Antimicrob Agents Chemother 52:3851–3862. 10.1128/AAC.00463-08. 18710914 PMC2573144

[28] Lee MD, Galazzo JL, Staley AL, Lee JC, Warren MS, Fuernkranz H, Chamberland S, Lomovskaya O, Miller GH. 2001. Microbial fermentation-derived inhibitors of efflux-pump-mediated drug resistance. Farmaco 56:81–85. 10.1016/s0014-827x(01)01002-3. 11347972

[29] Silva LV, Sanguinetti M, Vandeputte P, Torelli R, Rochat B, Sanglard D. 2013. Milbemycins: more than efflux inhibitors for fungal pathogens. Antimicrob Agents Chemother 57:873–886. 10.1128/AAC.02040-12. 23208712 PMC3553706

[30] Hiraga K, Yamamoto S, Fukuda H, Hamanaka N, Oda K. 2005. Enniatin has a new function as an inhibitor of Pdr5p, one of the ABC transporters in *Saccharomyces cerevisiae*. Biochem Biophys Res Commun 328:1119–1125. 10.1016/j.bbrc.2005.01.075. 15707993

[31] Shekhar-Guturja T, Tebung WA, Mount H, Liu N, Kohler JR, Whiteway M, Cowen LE. 2016. Beauvericin potentiates azole activity via inhibition of multidrug efflux, blocks *Candida albicans* morphogenesis, and is effluxed via Yor1 and circuitry controlled by Zcf29. Antimicrob Agents Chemother 60:7468–7480. 10.1128/AAC.01959-16. 27736764 PMC5119031

[32] Tanabe K, Lamping E, Nagi M, Okawada A, Holmes AR, Miyazaki Y, Cannon RD, Monk BC, Niimi M. 2011. Chimeras of *Candida albicans* Cdr1p and Cdr2p reveal features of pleiotropic drug resistance transporter structure and function. Mol Microbiol 82:416–433. 10.1111/j.1365-2958.2011.07820.x. 21895791

[33] Kueppers P, Gupta RP, Stindt J, Smits SH, Schmitt L. 2013. Functional impact of a single mutation within the transmembrane domain of the multidrug ABC transporter Pdr5. Biochemistry 52:2184–2195. 10.1021/bi3015778. 23464591

[34] Decottignies A, Grant AM, Nichols JW, de Wet H, McIntosh DB, Goffeau A. 1998. ATPase and multidrug transport activities of the overexpressed yeast ABC protein Yor1p. J Biol Chem 273:12612–12622. 10.1074/jbc.273.20.12612. 9575223

[35] Decottignies A, Kolaczkowski M, Balzi E, Goffeau A. 1994. Solubilization and characterization of the overexpressed *PDR5* multidrug resistance nucleotide triphosphatase of yeast. J Biol Chem 269:12797–12803. 10.1016/S0021-9258(18)99946-1. 8175692

[36] Lugo MR, Sharom FJ. 2014. Kinetic validation of the models for P-glycoprotein ATP hydrolysis and vanadate-induced trapping. Proposal for additional steps. PLoS One 9:e98804. 10.1371/journal.pone.0098804. 24897122 PMC4045855

[37] Urbatsch IL, Sankaran B, Weber J, Senior AE. 1995. P-glycoprotein is stably inhibited by vanadate-induced trapping of nucleotide at a single catalytic site. J Biol Chem 270:19383–19390. 10.1074/jbc.270.33.19383. 7642618

[38] Urbatsch IL, Tyndall GA, Tombline G, Senior AE. 2003. P-glycoprotein catalytic mechanism: studies of the ADP-vanadate inhibited state. J Biol Chem 278:23171–23179. 10.1074/jbc.M301957200. 12670938

[39] Rawal MK, Khan MF, Kapoor K, Goyal N, Sen S, Saxena AK, Lynn AM, Tyndall JD, Monk BC, Cannon RD, Komath SS, Prasad R. 2013. Insight into pleiotropic drug resistance ATP-binding cassette pump drug transport through mutagenesis of Cdr1p transmembrane domains. J Biol Chem 288:24480–24493. 10.1074/jbc.M113.488353. 23824183 PMC3750147

[40] Sauna ZE, Bohn SS, Rutledge R, Dougherty MP, Cronin S, May L, Xia D, Ambudkar SV, Golin J. 2008. Mutations define cross-talk between the N-terminal nucleotide-binding domain and transmembrane helix-2 of the yeast multidrug transporter Pdr5: possible conservation of a signaling interface for coupling ATP hydrolysis to drug transport. J Biol Chem 283:35010–35022. 10.1074/jbc.M806446200. 18842589 PMC2596398

[41] Golin J, Ambudkar SV, May L. 2007. The yeast Pdr5p multidrug transporter: how does it recognize so many substrates? Biochem Biophys Res Commun 356:1–5. 10.1016/j.bbrc.2007.02.011. 17316560

[42] Gupta RP, Kueppers P, Schmitt L, Ernst R. 2011. The multidrug transporter Pdr5: a molecular diode? Biol Chem 392:53–60. 10.1515/BC.2011.011. 21194365

[43] Shekhar-Guturja T, Gunaherath GM, Wijeratne EM, Lambert JP, Averette AF, Lee SC, Kim T, Bahn YS, Tripodi F, Ammar R, Dohl K, Niewola-Staszkowska K, Schmitt L, Loewith RJ, Roth FP, Sanglard D, Andes D, Nislow C, Coccetti P, Gingras AC, Heitman J, Gunatilaka AA, Cowen LE. 2016. Dual action antifungal small molecule modulates multidrug efflux and TOR signaling. Nat Chem Biol 12:867–875. 10.1038/nchembio.2165. 27571477 PMC5030160

[44] Golin J, Ambudkar SV. 2015. The multidrug transporter Pdr5 on the 25th anniversary of its discovery: an important model for the study of asymmetric ABC transporters. Biochem J 467:353–363. 10.1042/BJ20150042. 25886173 PMC4784962

[45] Golin J, Ambudkar SV, Gottesman MM, Habib AD, Sczepanski J, Ziccardi W, May L. 2003. Studies with novel Pdr5p substrates demonstrate a strong size dependence for xenobiotic efflux. J Biol Chem 278:5963–5969. 10.1074/jbc.M210908200. 12496287

[46] Ernst R, Kueppers P, Klein CM, Schwarzmueller T, Kuchler K, Schmitt L. 2008. A mutation of the H-loop selectively affects rhodamine transport by the yeast multidrug ABC transporter Pdr5. Proc Natl Acad Sci USA 105:5069–5074. 10.1073/pnas.0800191105. 18356296 PMC2278221

[47] Furman C, Mehla J, Ananthaswamy N, Arya N, Kulesh B, Kovach I, Ambudkar SV, Golin J. 2013. The deviant ATP-binding site of the multidrug efflux pump Pdr5 plays an active role in the transport cycle. J Biol Chem 288:30420–30431. 10.1074/jbc.M113.494682. 24019526 PMC3798506

[48] Kolaczkowski M, Sroda-Pomianek K, Kolaczkowska A, Michalak K. 2013. A conserved interdomain communication pathway of pseudosymmetrically distributed residues affects substrate specificity of the fungal multidrug transporter Cdr1p. Biochim Biophys Acta 1828:479–490. 10.1016/j.bbamem.2012.10.024. 23122779

[49] Saini P, Prasad T, Gaur NA, Shukla S, Jha S, Komath SS, Khan LA, Haq QM, Prasad R. 2005. Alanine scanning of transmembrane helix 11 of Cdr1p ABC antifungal efflux pump of *Candida albicans*: identification of amino acid residues critical for drug efflux. J Antimicrob Chemother 56:77–86. 10.1093/jac/dki183. 15937063

[50] Egner R, Bauer BE, Kuchler K. 2000. The transmembrane domain 10 of the yeast Pdr5p ABC antifungal efflux pump determines both substrate specificity and inhibitor susceptibility. Mol Microbiol 35:1255–1263. 10.1046/j.1365-2958.2000.01798.x. 10712705

[51] Egner R, Rosenthal FE, Kralli A, Sanglard D, Kuchler K. 1998. Genetic separation of FK506 susceptibility and drug transport in the yeast Pdr5 ATP-binding cassette multidrug resistance transporter. Mol Biol Cell 9:523–543. 10.1091/mbc.9.2.523. 9450972 PMC25282

[52] Harris A, Wagner M, Du D, Raschka S, Nentwig LM, Gohlke H, Smits SHJ, Luisi BF, Schmitt L. 2021. Structure and efflux mechanism of the yeast pleiotropic drug resistance transporter Pdr5. Nat Commun 12:5254. 10.1038/s41467-021-25574-8. 34489436 PMC8421411

[53] Lewinson O, Orelle C, Seeger MA. 2020. Structures of ABC transporters: handle with care. FEBS Lett 594:3799–3814. 10.1002/1873-3468.13966. 33098660 PMC7756565

[54] Spira F, Mueller NS, Beck G, von Olshausen P, Beig J, Wedlich-Soldner R. 2012. Patchwork organization of the yeast plasma membrane into numerous coexisting domains. Nat Cell Biol 14:640–648. 10.1038/ncb2487. 22544065

[55] van 't Klooster JS, Cheng TY, Sikkema HR, Jeucken A, Moody B, Poolman B. 2020. Periprotein lipidomes of *Saccharomyces cerevisiae* provide a flexible environment for conformational changes of membrane proteins. Elife 9:e57003. 10.7554/eLife.57003. 32301705 PMC7182430

[56] Al-Shawi MK, Polar MK, Omote H, Figler RA. 2003. Transition state analysis of the coupling of drug transport to ATP hydrolysis by P-glycoprotein. J Biol Chem 278:52629–52640. 10.1074/jbc.M308175200. 14551217

[57] Holmes AR, Tsao S, Ong SW, Lamping E, Niimi K, Monk BC, Niimi M, Kaneko A, Holland BR, Schmid J, Cannon RD. 2006. Heterozygosity and functional allelic variation in the *Candida albicans* efflux pump genes *CDR1* and *CDR2*. Mol Microbiol 62:170–186. 10.1111/j.1365-2958.2006.05357.x. 16942600

[58] Niimi K, Harding DR, Parshot R, King A, Lun DJ, Decottignies A, Niimi M, Lin S, Cannon RD, Goffeau A, Monk BC. 2004. Chemosensitization of fluconazole resistance in *Saccharomyces cerevisiae* and pathogenic fungi by a d-octapeptide derivative. Antimicrob Agents Chemother 48:1256–1271. 10.1128/AAC.48.4.1256-1271.2004. 15047528 PMC375246

[59] Clinical and Laboratory Standards Institute. 2008. M27-A3 reference method for broth dilution antifungal susceptibility testing of yeasts; approved standard, 3rd ed. Clinical and Laboratory Standards Institute, Wayne, PA.

[60] Holmes AR, Keniya MV, Ivnitski-Steele I, Monk BC, Lamping E, Sklar LA, Cannon RD. 2012. The monoamine oxidase A inhibitor clorgyline is a broad-spectrum inhibitor of fungal ABC and MFS transporter efflux pump activities which reverses the azole resistance of *Candida albicans* and *Candida glabrata* clinical isolates. Antimicrob Agents Chemother 56:1508–1515. 10.1128/AAC.05706-11. 22203607 PMC3294898

[61] Mukherjee PK, Sheehan DJ, Hitchcock CA, Ghannoum MA. 2005. Combination treatment of invasive fungal infections. Clin Microbiol Rev 18:163–194. 10.1128/CMR.18.1.163-194.2005. 15653825 PMC544182

[62] Odds FC. 2003. Synergy, antagonism, and what the chequerboard puts between them. J Antimicrob Chemother 52:1. 10.1093/jac/dkg301. 12805255

[63] Madani G, Lamping E, Cannon RD. 2021. Engineering a cysteine-deficient functional *Candida albicans* Cdr1 molecule reveals a conserved region at the cytosolic apex of ABCG transporters important for correct folding and trafficking of Cdr1. mSphere 6:e01318-20. 10.1128/mSphere.01318-20. 33568458 PMC8544900

[64] Madani G, Lamping E, Lee HJ, Niimi M, Mitra AK, Cannon RD. 2021. Small-scale plasma membrane preparation for the analysis of *Candida albicans* Cdr1-mGFPHis. J Vis Exp 172:e62592. 10.3791/62592 34180894

