# Supplementary material for: Inhibitor-Resistant Mutants Give Important Insights into Candida albicans ABC Transporter Cdr1 Substrate Specificity and Help Elucidate Efflux Pump Inhibition
Source: Antimicrob Agents Chemother. 2022 Jan 18;66(1):e01748-21. doi: 10.1128/AAC.01748-21 (PMC8765293; doi:10.1128/AAC.01748-21)
Supplement: Supplemental file 1 — Supplemental tables and figures. Download AAC.01748-21-s0001.pdf, PDF file, 1.4 MB [file aac.01748-21-s0001.pdf]

Supporting Information

Supporting Tables

Supporting Table S1. Mutations detected in the milbemycin α25-resistant AD/CDR1 isolates.

Isolate	*CDR1* mutation	Cdr1 mutation	Location
R25-1	G2137C	A713P	EL3
R25-2	G1561C	G521R	TMS1
R25-3	G1917C	M639I	TMS4
R25-5	C4064A	T1355N	TMS11
R25-6	G2137C	A713P	EL3
R25-7	G2137C	A713P	EL3
R25-8	G1917A	M639I	TMS4
R25-9	G1917A	M639I	TMS4

Supporting Table S2. Drug susceptibilities of AD/pABC3, AD/CDR1 and the strains overexpressing Cdr1 mutants.

Strain[1]	Azoles (mg/L)[2]							Other xenobiotics (mg/L)[2]					
	FLC 306	CLT 345	VRC 349	MCZ 416	KTC 531	PSC 701	ITC 706	CER 223	CHX 281	LaA 422	R6G 479	MON 681	NIG 724
pABC3	1.25	0.002	0.016	0.008	0.008	0.016	0.031	0.25	0.016	0.016	0.5	0.031	0.063
CDR1	640	8	8	8	8	32	32	8	2	8	32	128	128
G521R	160	0.002	1	0.5	0.125	0.063	0.25	4	1	0.125	4	0.5	0.25
M639I	640	8	8	2	4	2	16	4	1	8	16	128	128
A713P	320	1	4	1	1	1	1	4	1	2	32	128	8
T1355N	320	4	8	2	4	2	16	4	2	2	32	128	128

[1] pABC3 = AD/pABC3 (negative control); CDR1 = AD/CDR1 (positive control); G521R = AD/CDR1-G521R; M639I = AD/CDR1-M639I; A713P = AD/CDR1-A713P; T1355N = AD/CDR1-T1355N.

[2] Cdr1 drug substrates are listed according to their molecular weight from lowest (left) to highest (right). Numbers underneath each compound are the molecular weights in Da.

Supporting Table S3. Estimated IC_{50} values (μM)[1] for substrate inhibition of the ATPase activities of wt Cdr1 and the milbemycin α25 resistant G521R and A713P mutants.

	CLT	KTC	ITC	R6G	MON	NIG
CDR1	30	13	>32	32	>256	>256
G521R	>100	>1000	>32	32	none	>256
A713P	55	100	none[2]	>64	none	>256

[1] The estimated IC_{50} values were calculated from Supporting Fig. S4. No substrate inhibited the ATPase activity by more than ~75%.

[2] None means even the highest substrate concentrations did not reduce the ATPase activity at all; values with larger than (>) signs indicate only partial inhibition of the ATPase activity (i.e. the ATPase activities could not be inhibited by 50% or more even at highest possible test concentrations).

Supporting Table S4. Mutations detected in the enniatin B resistant AD/CDR1 isolates.

Isolate	*CDR1* mutation	Cdr1 mutation	Location
EN-1	G1561C	G521R	TMS1
EN-2	G1562T	G521V	TMS1
EN-3	G1917A	M639I	TMS4
EN-4	T1990A	L664I	TMS5
EN-5	T1994C	L665S	TMS5
EN-6	G2002A	V668I	TMS5
EN-7	T3703G	F1235V	TMS8
EN-8	G4068T	M1356I	TMS11

Supporting Table S5. Mutations detected in the beauvericin resistant AD/CDR1 isolates.

Isolate	*CDR1* mutation	Cdr1 mutation	Location
BE-1	G1561A	G521S	TMS1
BE-2	G1561T	G521C	TMS1
BE-3	G1562T	G521V	TMS1
BE-4	T2003A	V668D	TMS5

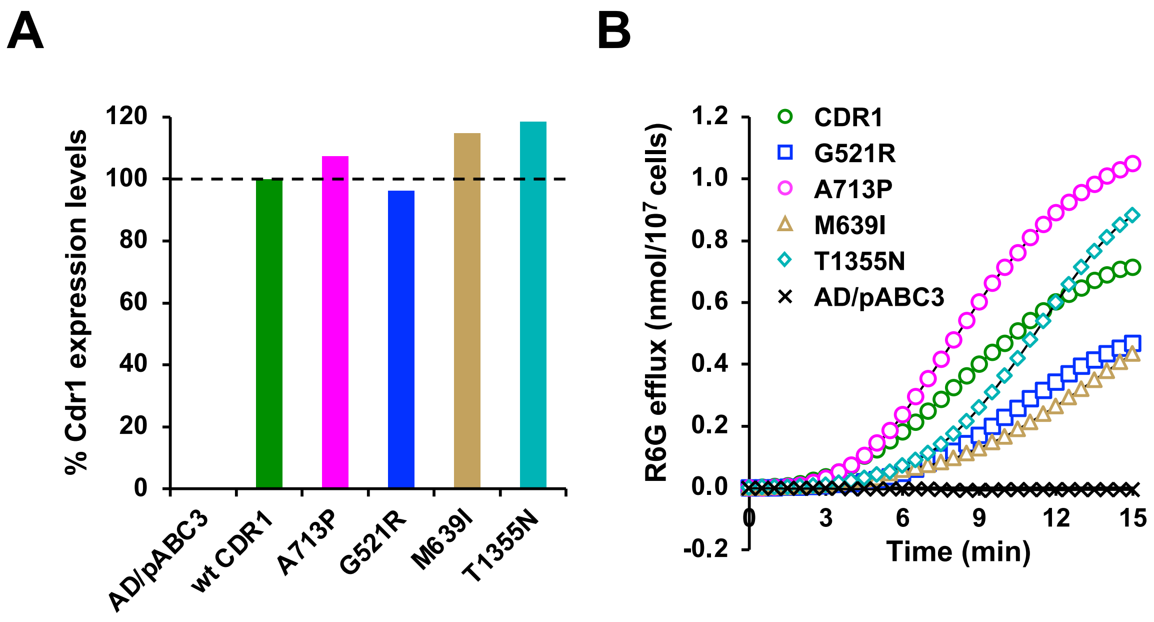

Supporting Figure S1. Expression levels and R6G efflux pump activities of wt Cdr1 and the four milbemycin α25 resistant Cdr1 variants. (A) The intensities of the coomassie-blue stained Cdr1 plasma membrane protein bands in Fig. 1B were quantified with ImageJ and normalized to wt Cdr1. (B) Graph of the time dependent R6G efflux pump activities of 10^7 of the indicated mid-log phase cells; cells were pre-loaded for 90 min with 15 μM R6G in the presence of 2-deoxy-glucose and, after washing, the cells were resuspended in 50 mM HEPES buffer (pH 7.0) and R6G efflux was initiated by the addition of 5 mM glucose. No R6G efflux pump activity was detected for AD/pABC3 or for any of the Cdr1 overexpressing strains in the absence of glucose (data not shown). The results are the means of two independent experiments.

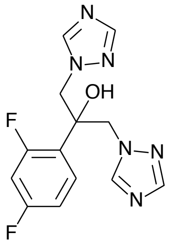

FLC
(306.27)

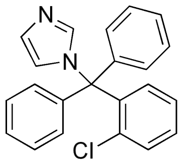

CLT
(344.84)

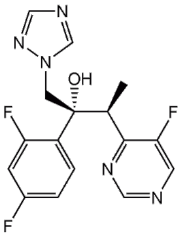

VRC
(349.31)

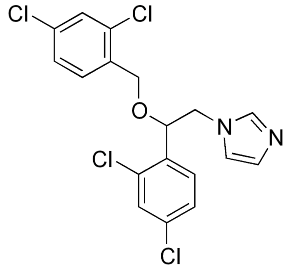

MCZ
(416.13)

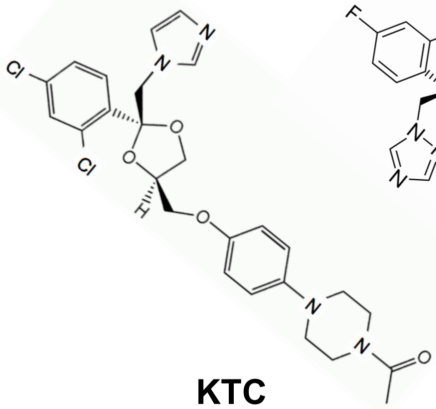

KTC
(531.43)

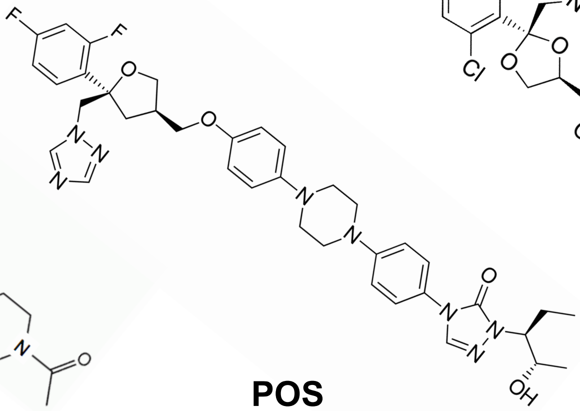

POS
(700.78)

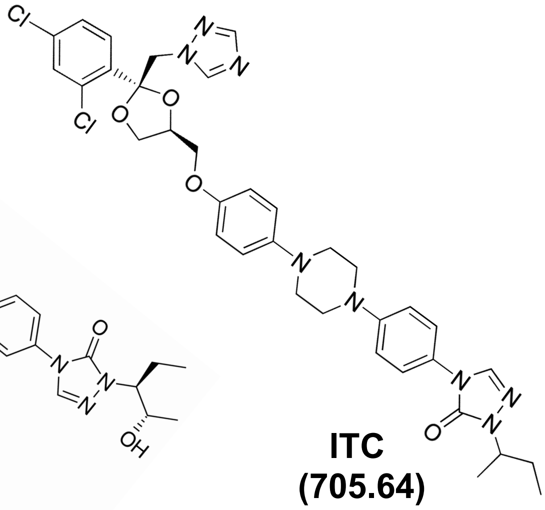

ITC
(705.64)

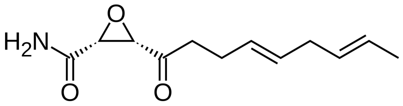

CER
(223.27)

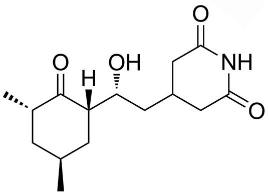

CHX
(281.35)

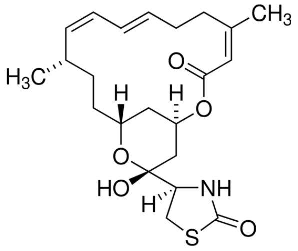

LaA
(421.55)

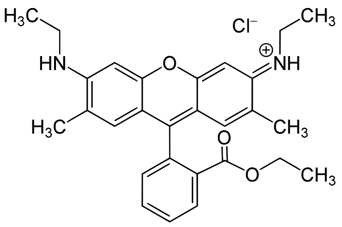

R6G
(479.02)

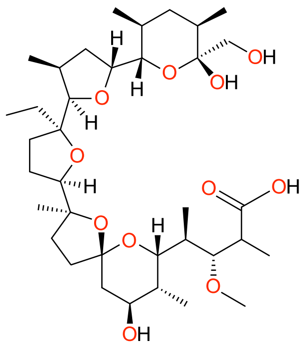

MON
(680.87)

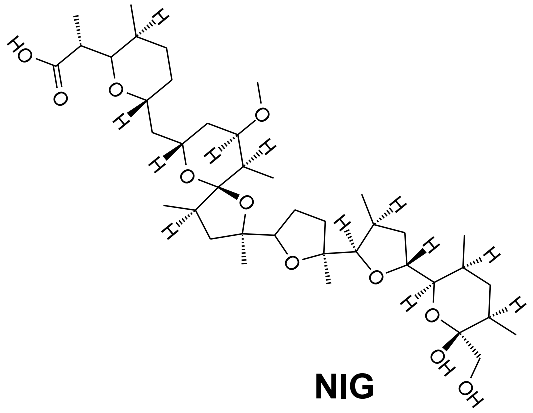

NIG
(724.96)

Supporting Figure S2. Cdr1 substrates with their MWs in brackets (Da).

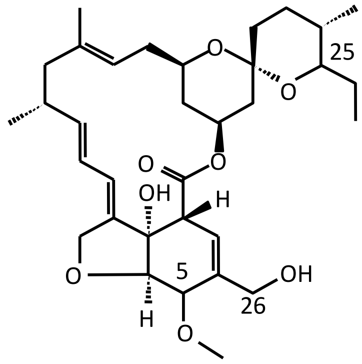

milbemycin α25
(572.0)

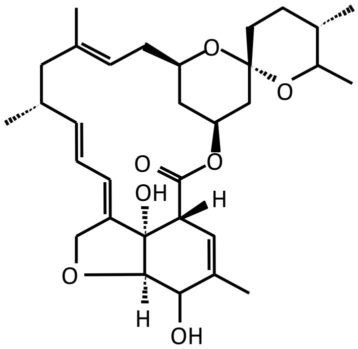

milbemycin A3
(528.7)

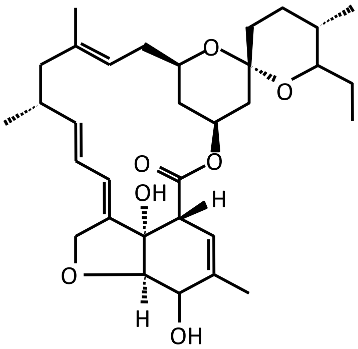

milbemycin A4
(542.3)

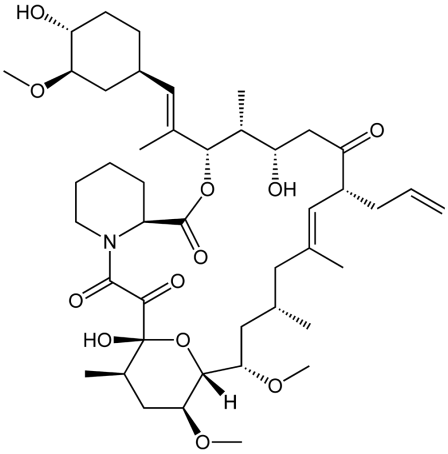

FK506
(804.0)

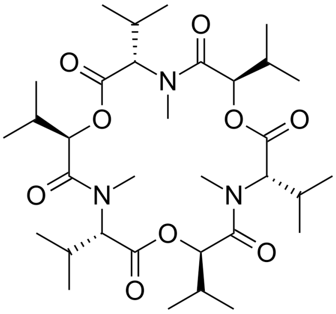

enniatin B
(639.8)

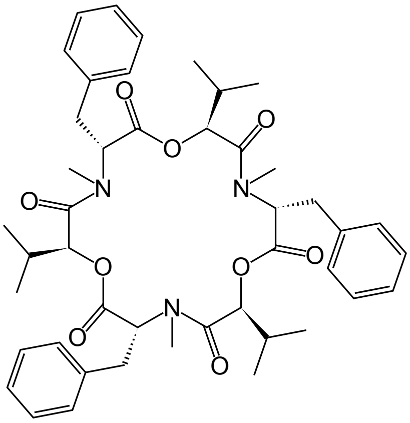

beauvericin
(784.0)

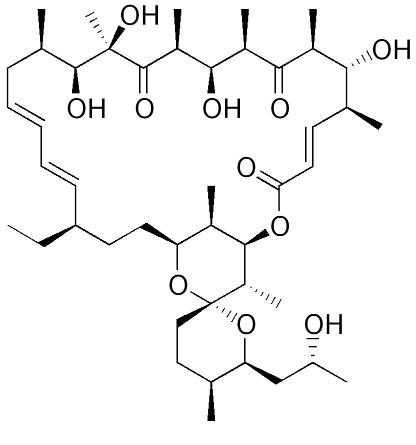

oligomcyin
(791)

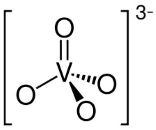

orthovanadate
(114.9)

Supporting Figure S3. Cdr1 efflux pump inhibitors with their MWs in brackets (Da).

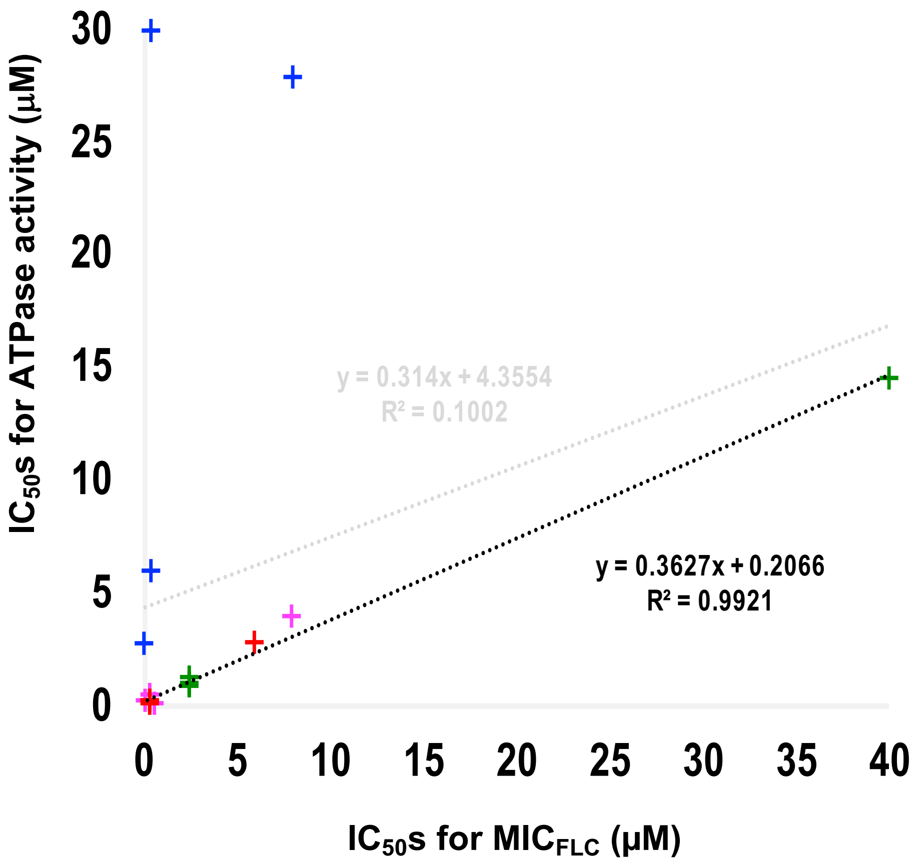

Supporting Figure S4. There was a good correlation ($R^2 = 0.99$; black dotted trendline) between the IC_{50} values for the inhibition of the ATPase activities of isolated plasma membranes (y-axis) and the FLC efflux pump inhibition of intact cells (x-axis) by FK506 (green), enniatin B (magenta) and beauvericin (red) in wt Cdr1, G521R, M639I and T1355N overexpressing cells, but only if the data for milbemycin α25 (blue) were excluded from the calculations (grey dotted trendline; $R^2 = 0.10$). The data were extracted from Table 4.

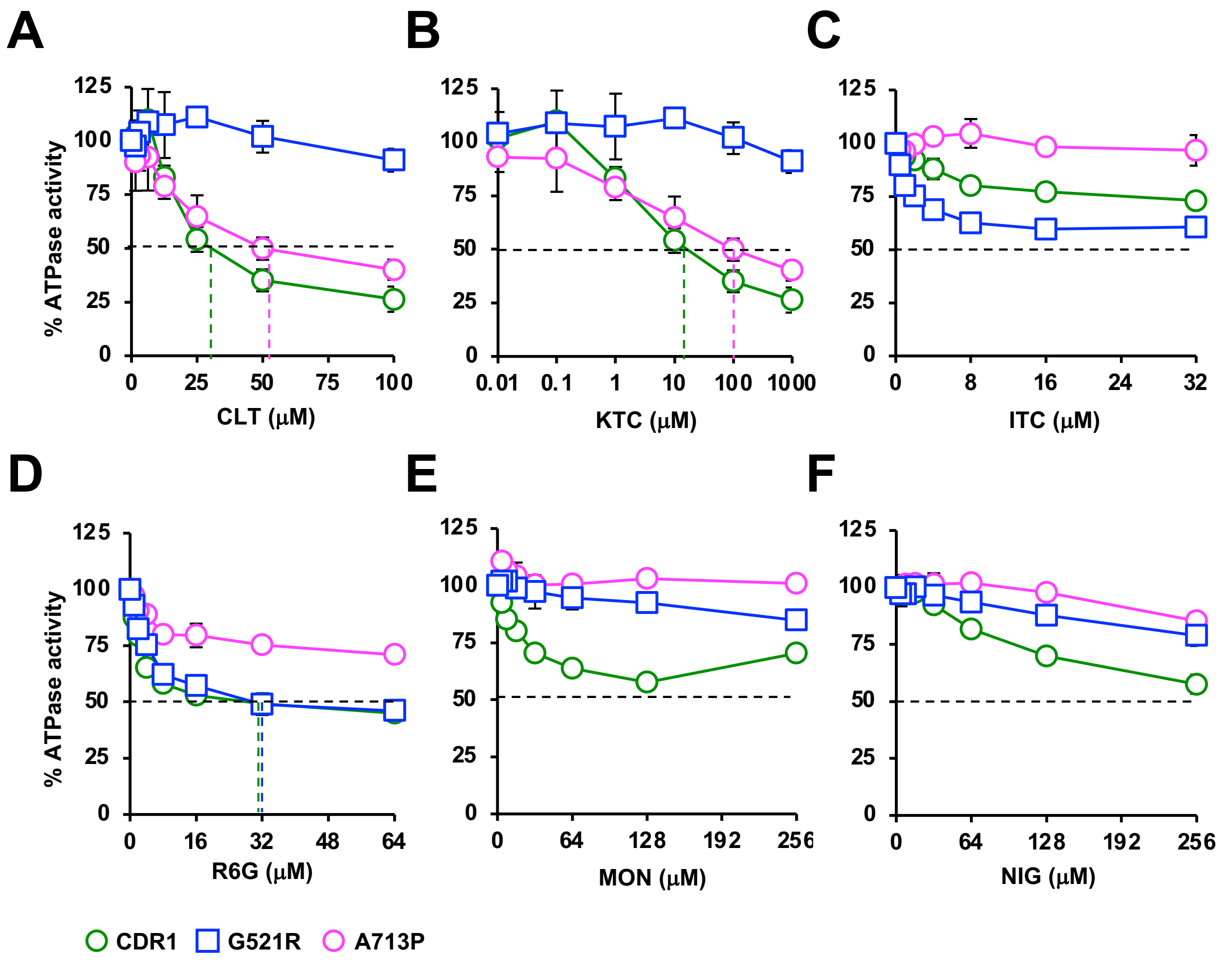

Supporting Figure S5. Effect of substrates on the ATPase activities of wt Cdr1 and the milbemycin α25-resistant mutants G521R and A713P. The mean ATPase activities of three independent experiments (± SD) were expressed as the percentage of the uninhibited ATPase activities. The ATPase activities of wt Cdr1 (green circles), Cdr1-G521R (blue squares), and Cdr1-A713P (pink circles) in response to increasing concentrations of CLT (A), KTC (B), ITC (C), R6G (D), MON (E) and NIG (F) are shown. Dashed horizontal lines indicate 50% inhibition and coloured vertical dashed lines the IC_{50} values for the corresponding substrate.

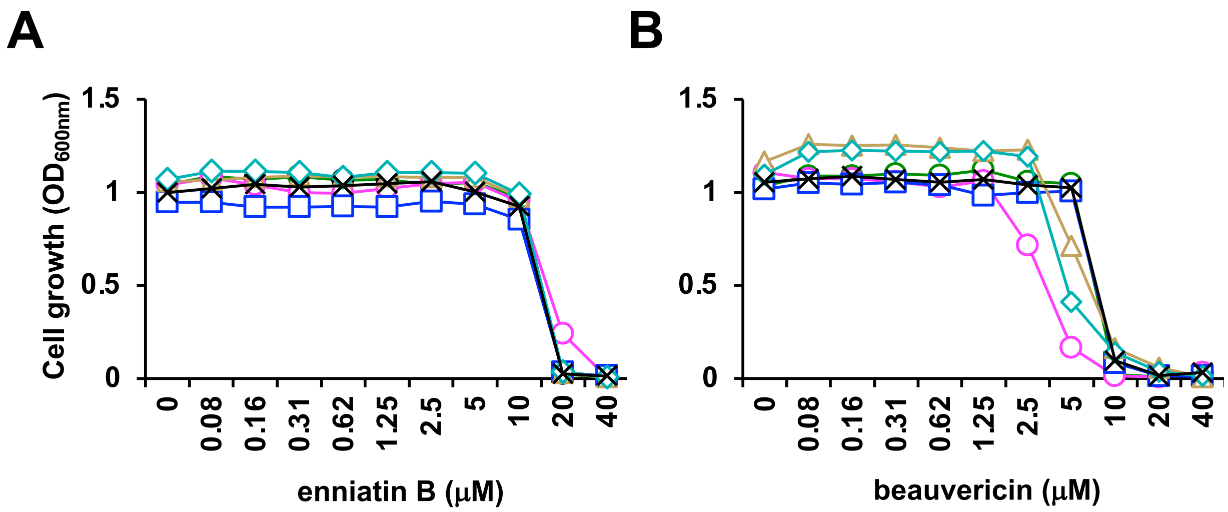

Supporting Figure S6. Pump inhibitor susceptibilities of the sensitive control strain AD/pABC3 and AD1-8u$^-$ cells overexpressing wt Cdr1 and the milbemycin α25 resistant Cdr1 mutants. The enniatin B and beauvericin susceptibilities of cells grown in CSM medium adjusted to pH 7.0 were determined after 2 days of incubation with shaking at 150 rpm at 30˚C. (A) Enniatin B susceptibilities and (B) beauvericin susceptibilities. x, AD/pABC3; ○, CDR1; ○, A713P; □, G521R; △, M639I; ◇, T1355N.
